# MicroRNA-143 is a putative predictive factor for the response to fluoropyrimidine-based chemotherapy in patients with metastatic colorectal cancer

**DOI:** 10.18632/oncotarget.4035

**Published:** 2015-05-08

**Authors:** Femke Simmer, Sabine Venderbosch, Jeroen R. Dijkstra, Elisa M. Vink-Börger, Claudius Faber, Leonie J. Mekenkamp, Miriam Koopman, Anton F. De Haan, Cornelis J. Punt, Iris D. Nagtegaal

**Affiliations:** ^1^ Department of Pathology, Radboud University Medical Center, Nijmegen, The Netherlands; ^2^ Department of Medical Oncology, Academic Medical Center, University of Amsterdam, Amsterdam, The Netherlands; ^3^ Institute of Pathology, Ludwig-Maximilians-University of München, München, Germany; ^4^ Department of Medical Oncology, University Medical Center Utrecht, Utrecht, The Netherlands; ^5^ Department for Health Evidence, Section Biostatistics, Radboud University Medical Center, Nijmegen, The Netherlands

**Keywords:** microRNA, biomarker, chemotherapy, fluoropyrimidine, colorectal cancer

## Abstract

Approximately half of the colorectal cancer (CRC) patients develop metastatic disease. Fluoropyrimidine-based chemotherapy forms the backbone of treatment in these patients. However, the response to this therapy varies between individuals. Therefore, an important challenge in CRC research is to identify biomarkers that are predictive of this response. In this study, we explored the potential of miRNAs, and the miRNA producing protein Dicer, as biomarkers that can predict chemo-sensitivity to fluoropyrimidine chemotherapy in patients with metastatic colorectal cancer (mCRC). We analyzed the levels of 22 miRNAs and the Dicer protein in primary tumors from patients with mCRC who were treated with first-line capecitabine monotherapy within the CAIRO trial of the Dutch Colorectal Cancer Group. Correlation between the expression status of miRNAs or Dicer in primary tumors and the progression free survival (PFS) were investigated. Patients with low expression of miR-143 in their primary tumor had increased median PFS compared to those with high expression of miR-143. Furthermore, FXYD3, an ion transport regulator and a putative target of miR-143, also showed an association with PFS. These findings warrant further studies to investigate the relationship between miR-143, FXYD3 and fluoropyrimidines, and the clinical utility of miR-143 as biomarker.

## INTRODUCTION

Colorectal cancer (CRC) is worldwide one of the most common types of cancer, and a leading cause of cancer death for both men and women (http://globocan.iarc.fr). Approximately half of the CRC patients develop metastatic disease, either at diagnosis or during follow-up [1]. For over 40 years fluoropyrimidine-based chemotherapy (5-fluorouracil, capecitabine) has been used in the treatment of patients with metastatic CRC (mCRC), and significantly prolongs survival [2]. However, not all patients respond to treatment [3]. Moreover, chemotherapy is associated with toxicity. Therefore, biomarkers that can differentiate patients into responders and non-responders would optimize health care.

Much effort has already been put into the development of molecular markers predicting the response to fluoropyrimidine-based chemotherapy. Thymidylate synthase (TS) is possibly the most extensively studied biomarker in this context. However, for the metastatic setting none of the studied markers is ready for implementation in daily clinical practice [4].

MicroRNAs (miRNAs) are non-coding RNAs that inhibit gene expression post-transcriptionally. They are encoded within the genome and are initially transcribed as large primary transcripts (several kilobases) by RNA polymerase II. Two RNase III enzymes successively cleave the primary transcripts, Drosha in the nucleus and Dicer in the cytoplasm. Ultimately, a 22 nt long single-stranded mature miRNA is incorporated into the multi-protein RNA-induced silencing complex (RISC), which as a whole binds to the 3′ untranslated region of a target messenger RNA (mRNA). Imperfect binding to target mRNA represses its translation, whereas perfect binding leads to cleavage and degradation of the target mRNA [5].

MiRNAs influence basic biological processes such as growth, invasion, proliferation, differentiation, angiogenesis and cell death. The effect of one specific miRNA can be widespread, since it can potentially modulate hundreds of different downstream genes. Altered miRNA levels are implicated in early tumorigenesis as well as disease progression. Moreover, there are indications that they can affect chemo-sensitivity of cancer cells [6–13]. In combination with their remarkable stability that allows detection in formalin-fixed, paraffin-embedded (FFPE) material [14], miRNAs are molecules that could serve as biomarkers for chemotherapy.

This study was designed to identify a predictive factor for the response to fluoropyrimidine chemotherapy in patients with mCRC, focusing at the miRNA pathway. More specifically, we analyzed the expression of Dicer and 22 miRNAs that were shown to be associated with CRC.

## RESULTS

### Assessment of associations between Dicer and survival

**Figure 1:**
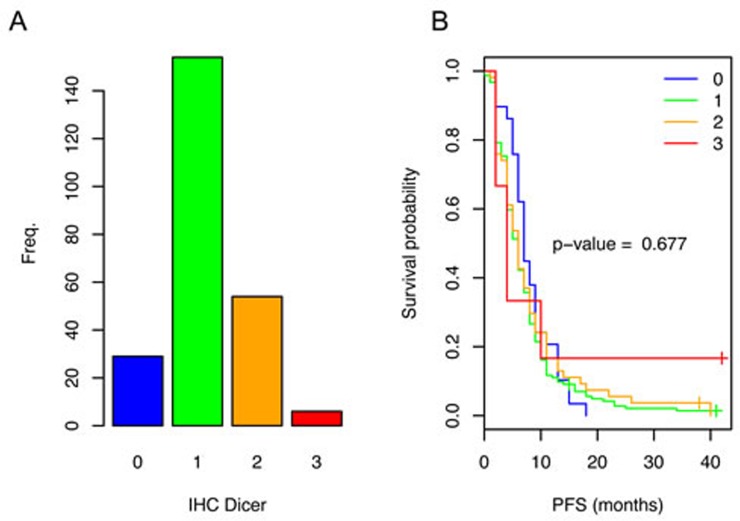
Dicer expression in the primary tumors of patients with mCRC and the related PFS **A.** Variable Dicer staining intensities were observed (IHC0-3). The number of patients per category of Dicer staining are depicted in a bar plot. **B.** Kaplan-Meier survival analysis comparing CRC patients with different Dicer staining intensities in the tumor. No significant association between PFS and Dicer staining was observed.

Immunohistochemical (IHC) analysis was performed to study the influence of Dicer expression on progression free survival (PFS) of mCRC patients who received fluoropyrimidine-based chemotherapy. Of the 243 analyzed tumors (Supplemental Table 1 contains the baseline characteristics of the patients and tumors), only six tumors showed strong Dicer staining (IHC 3), whereas most (*n* = 154) showed a weak staining (IHC 1) (Figure 1A). Survival analysis for this study population did not show a significant association between PFS and Dicer staining (Figure 1B).

### Comparison of miRNA expression in primary tumors and matched normal tissue

We next investigated the predictive potential of miRNAs for patients with mCRC who received fluoropyrimidine-based chemotherapy. The miRNAs were selected based on literature research for studies linking miRNAs to (fluoropyrimidine-based) chemotherapy and/or CRC. In total, we analyzed the mature miRNA levels of 22 miRNA using stem-loop quantitative reverse transcriptase-polymerase chain reaction (Supplemental Table 4).

**Figure 2:**
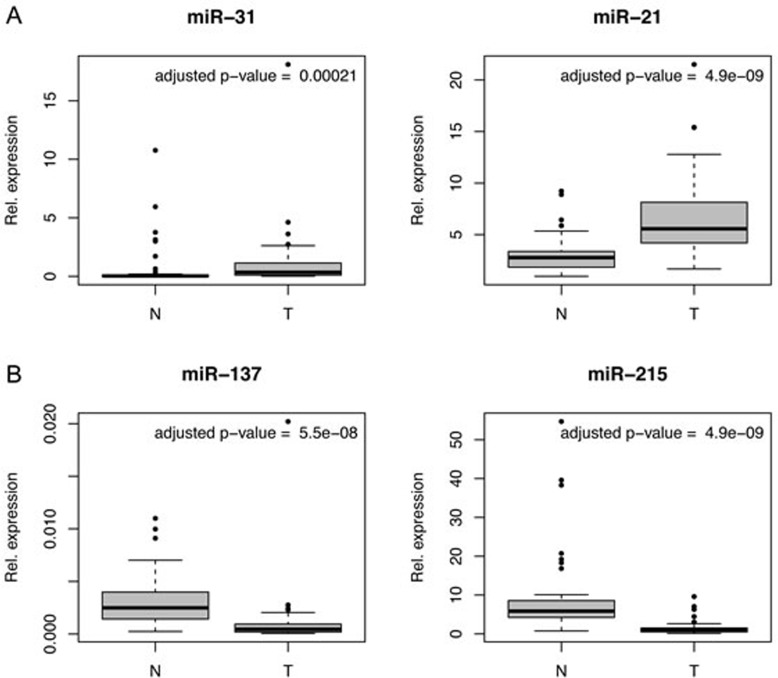
MiRNA expression in CRC tumors (T) compared to normal (N) mucosa Box plots for four exemplary miRNAs displaying differences between the expression in tumor and normal mucosa. **A.** miR-31 and miR-21 showed a higher median expression in the tumor. **B.** miR-137 and miR-215 showed lower median expression in the tumor.

For 55 patients we assessed the relative miRNA levels in tumor and normal tissue. For five miRNAs (miR34a, miR-93, miR-19b, miR-92a, miR125b) no significant difference between expression in tumor and normal tissue was observed (P > 0.05), for the others there was a significant difference (Table 1). Especially, miR-31 and miR-21 showed a higher median expression in tumor tissue, and miR-137 and miR-215 showed lower median expression in tumor compared to normal tissue (Table 1 and Figure 2).

**Table 1:** Comparison of the miRNA expression in tumor and normal mucosa

Expression analysis: normal vs tumor							
	miRNA	Median Expression Tumor	Median Expression Normal	T/N	*p*-value Wilcoxon test	Adjusted *p*-value Wilcoxon test	
**1**	**miR-16**	0.709	1.363	0.520	3.58E-10	4.87E-09	
**2**	**miR-18a**	0.047	0.035	1.361	7.23E-04	9.94E-04	
**3**	**miR-19b**	4.032	3.963	1.017	6.78E-01	7.46E-01	*
**4**	**miR-21**	5.574	2.779	2.005	8.85E-10	4.87E-09	
**5**	**miR-26b**	0.137	0.314	0.437	5.49E-10	4.87E-09	
**6**	**miR-31**	0.348	0.033	10.432	1.45E-04	2.13E-04	
**7**	**miR-34a**	0.257	0.198	1.297	5.66E-02	6.92E-02	*
**8**	**miR-92a**	0.454	0.400	1.134	7.34E-01	7.69E-01	*
**9**	**miR-93**	0.262	0.313	0.838	1.71E-01	1.98E-01	*
**10**	**miR-103**	0.136	0.229	0.593	2.24E-08	5.47E-08	
**11**	**miR-125b**	0.376	0.419	0.897	9.30E-01	9.30E-01	*
**12**	**miR-137**	0.000	0.002	0.181	2.13E-08	5.47E-08	
**13**	**miR-140**	0.116	0.182	0.635	6.31E-05	1.07E-04	
**14**	**miR-143**	1.853	3.509	0.528	1.22E-04	1.92E-04	
**15**	**miR-145**	9.010	26.988	0.334	6.06E-07	1.21E-06	
**16**	**miR-148a**	0.076	0.118	0.643	3.79E-03	4.91E-03	
**17**	**miR-191**	0.757	1.553	0.488	2.04E-09	6.40E-09	
**18**	**miR-192**	1.197	3.882	0.308	1.42E-09	5.77E-09	
**19**	**miR-215**	0.882	5.849	0.151	8.85E-10	4.87E-09	
**20**	**miR-222**	0.812	1.251	0.649	5.68E-05	1.04E-04	
**21**	**let7a**	0.449	0.869	0.516	2.00E-07	4.41E-07	
**22**	**let7g**	0.283	0.550	0.514	1.57E-09	5.77E-09	

* Indicates > 0.05

**Table 2:** Comparison of the survival distributions of patients sub-grouped based on miRNA expression

Survival analysis: PFS for patients with low vs high miRNA expressing tumors			
	miRNA	p-value Log-rank test	adjusted p-value Log-rank test
**1**	**miR-16**	0.095	0.249
**2**	**miR-18a**	0.524	0.576
**3**	**miR-19b**	0.468	0.544
**4**	**miR-21**	0.329	0.483
**5**	**miR-26b**	0.995	0.995
**6**	**miR-31**	0.276	0.433
**7**	**miR-34a**	0.196	0.376
**8**	**miR-92a**	0.040	0.219
**9**	**miR-93**	0.023	0.168
**10**	**miR-103**	0.160	0.351
**11**	**miR-125b**	0.055	0.219
**12**	**miR-137**	0.060	0.219
**13**	**miR-140**	0.856	0.897
**14**	**miR-143**	0.001	0.012
**15**	**miR-145**	0.100	0.249
**16**	**miR-148a**	0.390	0.536
**17**	**miR-191**	0.205	0.376
**18**	**miR-192**	0.102	0.249
**19**	**miR-215**	0.009	0.095
**20**	**miR-222**	0.470	0.544
**21**	**miR-let7a**	0.464	0.544
**22**	**miR-let7g**	0.244	0.413

### Evaluation of correlations between Dicer staining and miRNA levels

**Figure 3:**
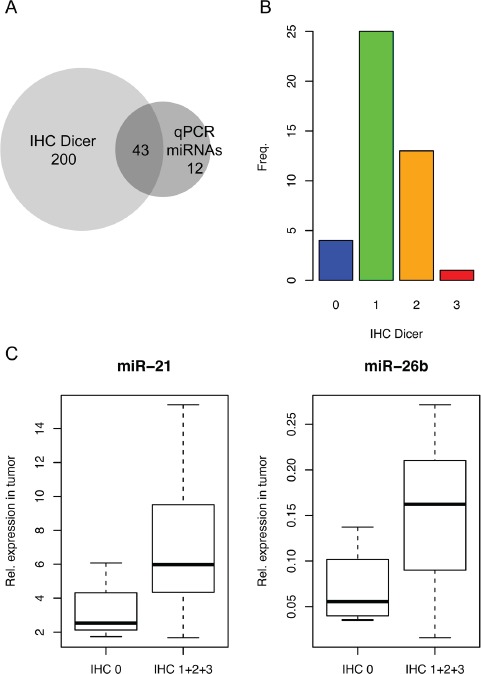
Evaluation of correlations between Dicer staining and miRNA levels **A.** Heat map indicating the correlation between Dicer expression and miRNA level. Positive correlations are black; negative correlations are white. Small p-values are in red; *p*-values above 0.05 are in pink. **B.** miR-21 and miR-26b showed higher expression in the samples with Dicer expression compared to those without.

For 43 patients we collected both IHC for Dicer as well as miRNA expression data (Figure 3A). We used this to investigate if the Dicer staining correlated with the miRNA expression level data. We compared tumors with and without staining for Dicer (IHC 0 = 4; IHC 1+2+3 = 39 (Figure 3B). Only, miR-21 and miR-26b showed the expected positive correlation between Dicer expression and miRNA level (Figure 3C).

### MiR-143 is a putative predictive biomarker

**Figure 4:**
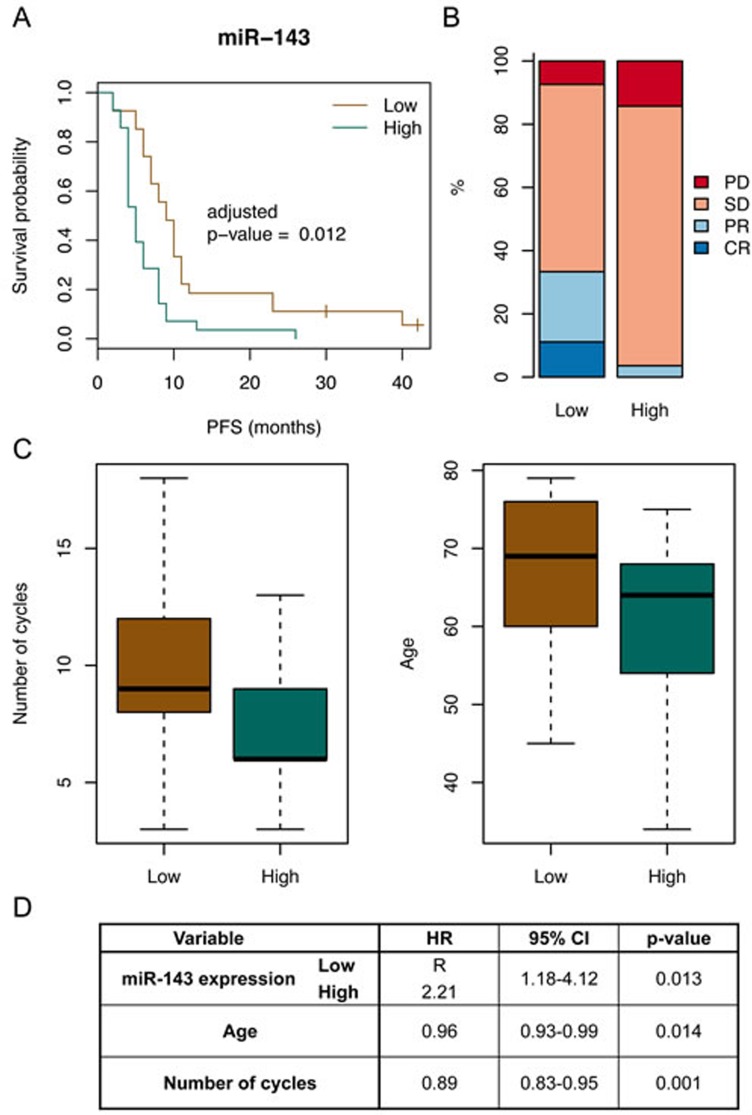
MiR-143 expression is associated with PFS **A.** Survival curves for patients with low and high miR-143 expression in their primary tumor (log rank test adjusted *p*-value = 0.012). High expression of miR-143 was also associated with a shorter PFS. **B.** Bar graphs for patients with low and high miR-143 expression in their primary tumor showing the proportions of response to therapy evaluated according to the RECIST criteria (fisher test *p*-value = 0.037). Complete response (CR); partial response (PR); no change/stable disease (S**D.**; progressive disease (P**D.**. The proportions of patients that experienced response to therapy (CR or PR) were higher for patients with primary tumors with low miR-143 expression levels as compared to primary tumors with high miR-143 expression levels. **C.** Distribution of other variables in the two sub-groups based on miR-143 expression. Left: distributions of the number of cycles of capecitabine that were administered to the patients (Kruskal-Wallis test *p*-value = 0.012). Right: distributions of the age (in years) of the patients at diagnosis (Kruskal-Wallis test *p*-value = 0.007). **D.** Table showing the results of the multivariate Cox regression analysis. MiR-143 expression is the strongest independent predictors of PFS.

To determine whether any of the 22 miRNAs were associated with PFS, the patients were sub-divided based on the median expression of the miRNA in the tumor tissues, Kaplan-Meier curves were made, log-rank test performed, and we adjusted for multiple testing. This approach showed in particular a significant difference for miR-143 (Table 2). The median PFS of the sub-groups (median PFS for low expression = 9 months (95%CI: 7-11); median PFS for high expression = 5 months (95%CI: 4-8)) and the Kaplan-Meier curves suggest that low miR-143 expression in tumor tissue is associated with increased PFS (adjusted *p*-value = 0.012, Figure 4A). There was no significant difference observed for OS, but when looking at response, as defined based on RECIST criteria, there was also a significant difference, with more response in the sub-group with low miR-143 expression (*p*-value = 0.037, Figure 4B). We subsequently checked if other clinico-pathological characteristics could play a role as confounders. We observed that, in the sub-group with low miR-143 expression and increased PFS, the median age of the patients at diagnosis was somewhat higher and that the number of cycles of capecitabine that were administered was higher (Supplemental Table 2 and Figure 4C). Finally, in multivariate Cox regression analysis (using only the variables that showed a significant difference in univariate analysis) miR-143 expression was the strongest predictor of PFS (Figure 4D).

### Prediction of target genes

One miRNA can regulate many different target genes, and in principle a small change in miRNA level could have a large biological effect. To get insight in how miR-143 and sensitivity to fluoropyrimidines are linked, two web-based prediction algorithms were used to identify putative target genes. Both computations generated a substantial list of candidates (Material and methods, Supplemental Table 5 and 6).

In addition, TCGA miRNA-seq and RNA-seq data were analyzed to select putative target genes. We assumed that miR-143 degrades its target mRNAs, and selected those genes that showed a negative correlation with miR-143 expression (Supplemental Table 7).

**Figure 5:**
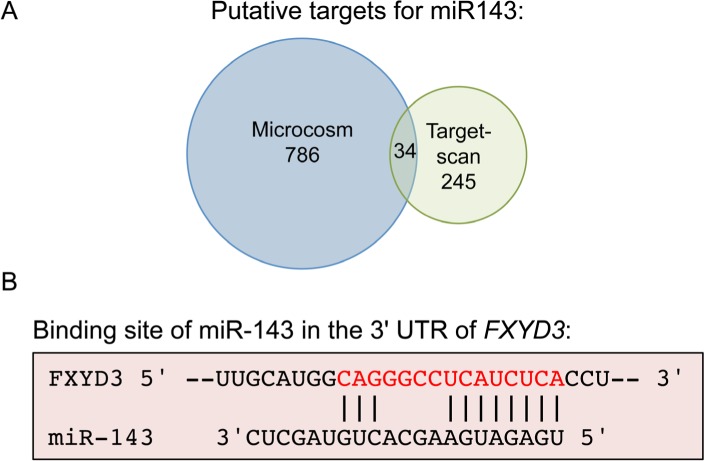
FXYD3 is putative target of miR-143 The web-based prediction algorithms MicroCosm and TargetScan were used to identify putative miR-143 target genes. **A.** Venn diagram depicting the number of overlapping and unique genes. **B.** Possible binding site of miR-143 in the 3′ UTR of *FXYD3*.

Next, overlaps between the gene lists obtained with the different approaches were determined. Thirty-four genes were common in the lists obtained with the target prediction algorithms (Figure 5A). From this overlap the gene FXYD3 was the only gene that also showed a negative correlation. This is a protein that belongs to a small family of proteins that can regulate sodium-potassium pumps, with one possible binding site of miR-143 in its 3′ UTR (Figure 5B).

### Expression of FXYD3

**Figure 6:**
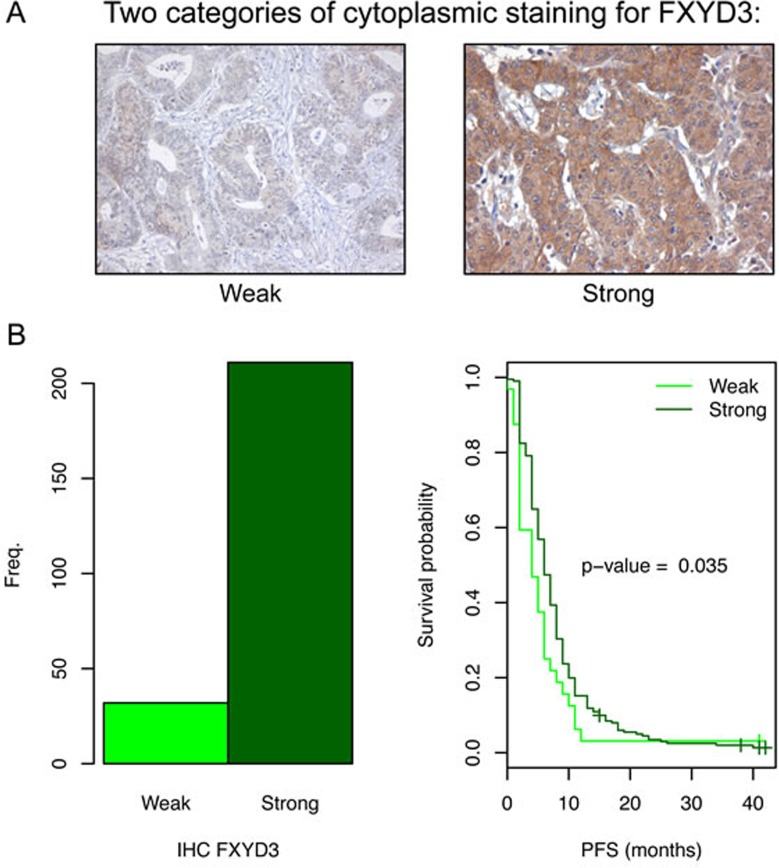
FXYD3 expression in the primary tumors of patients with mCRC and the related PFS **A.** Two different tumor samples with different cytoplasmic staining intensity for FXYD3 (weak vs strong). **B.** Two patient sub-groups based on FXYD3 expression. Left: the number of patients per staining category. The majority of the samples showed strong staining. Right: Kaplan-Meier survival analysis comparing patients with weak and strong staining. A significant association between PFS and FXYD3 was observed.

IHC analysis was performed to check if FXYD3 expression correlated with the miR-143 expression, and to study the influence of FXYD3 expression on PFS of mCRC patients who received fluoropyrimidine-based chemotherapy. We observed cytoplasm, membrane and nuclear staining. In the cytoplasm weak and strong staining was distinguished (Figure 6A).

The same 243 patients were analyzed as for the Dicer protein. Unfortunately, we could not properly analyze the correlation between FXYD3 and miR-143 expression, because only very few tumors showed the weak staining. Among the 43 patients for which we collected both IHC for FXYD3 and miR-143 expression data there were only 2 tumors with low FXYD3 expression in the cytoplasm compared to 41 tumors with high FXYD3 expression.

Of all tumors with IHC for FXYD3, 32 tumors showed weak, whereas 211 showed a strong cytoplasmic staining (Figure 6B). Interestingly, survival analysis showed a significant association between PFS and FXYD3 staining in the cytoplasm (Figure 6B). The median PFS for patients with weak and strong staining was 4 months (95%CI: 2-6) and 6 months (95%CI: 6-7), respectively. Thus, for FXYD3 higher expression appears associated with increased PFS, which is as expected, opposite to the results for miR-143.

Next, we checked if other clinico-pathological features could play a role as confounders. For all of the analyzed characteristics there was no significant difference between the sub-groups with FXYD3 weak and strong staining, except for histology and differentiation grade of the primary tumor. Of the 32 tumors with FXYD3 weak staining, there were two tumors that were classified as undifferentiated carcinoma, whereas there was no tumor with this classification in the 211 tumors with strong staining (Supplementary Table 3). Multivariate Cox regression analysis showed that both FXYD3 staining and differentiation grade were independent predictors for PFS (HR 1.51; 95%CI 1.03-2.21 and HR 1.08; 95%CI 1.02-1.15, respectively).

## DISCUSSION

A biomarker that allows stratifying patients into a sub-group that will respond and a subgroup that most likely does not benefit from the therapy, will contribute to personalized medicine. In this tissue-based study we have tested Dicer and several relevant miRNAs for their predictive value for the response to fluoropyrimidine-based chemotherapy in patients with mCRC. Among the selected patients we found that miR-143 could be used to identify a sub-group that had more benefit from fluoropyrimidine treatment.

Dicer expression, and its association to overall survival, was already studied in several CRC patient populations. For patients with stage II CRC, low Dicer expression was shown to be associated with improved survival [15]. In addition, for a group of patients with mCRC, low Dicer expression was also associated with better survival [16]. In contrast, in a study with primary tumors of all stages no relationship between Dicer expression and survival was found [17]. Similar to the other studies, we observed variable levels of Dicer expression in CRC. However, the Dicer expression could not be linked to PFS of patients with mCRC treated with fluoropyrimidine-based chemotherapy.

Furthermore, even though Dicer is a central enzyme in miRNA processing, in this study there was no clear correlation between miRNA and Dicer expression for most miRNAs. In the study by Stratmann et al (2011), who used qPCR to measure Dicer levels, there was only a positive correlation between Dicer expression and miRNA expression for one out of four miRNAs [17]. An explanation could be that, even though Dicer was not detected, there was still a low level of Dicer that can maintain the miRNA levels. On the other hand, it is also likely that more factors are involved in the biogenesis and maintenance of miRNA levels. For example, in zebrafish and mice Argonaute2, instead of Dicer, processes miRNA-451 [18, 19].

We observed for 17 out of 22 miRNAs a significant difference in expression between tumor and normal tissue (Table 1). Our data is consistent with published studies, in particular for miR-21 [18, 39], miR-137 [41, 42] and miR-18a [40]. The main aim of this study was to identify predictive markers for the response to fluoropyrimidine-based therapy in patients. Reports on cell line experiments and sometimes xenografts suggested that expression of miRNAs influences therapeutic efficacy of 5-fluorouracil, but patient data were generally lacking. Per miRNA, the association between expression level and PFS was assessed. For most miRNAs we did not observe a significant relationship. This could be in part because the analyzed sample set was too small and/or too homogeneous with relatively good prognostic characteristics.

For miR-143 we observed a significant association between its expression level and PFS. The group with low miR-143 expression showed longer PFS, suggesting higher sensitivity to capecitabine. Multivariate analysis also indicated that miR-143 could be a strong independent marker for response to capecitabine. In contrast, Borralho et al (2009) showed that over-expression of miR-143 in the colorectal cell line HCT116 resulted in increased sensitivity to 5-fluorouracil [20]. This does not fit with what we observed. However, since miR-143 targets many genes that each have their own transcriptional regulation, it could be that the function of miR-143 differs under different circumstances (e.g. *in vivo vs in vitro*; early vs late tumor stage; 5-fluorouracil vs capecitabine).

In an attempt to explain the link between miR-143 down-regulation and increased PFS, putative target genes were identified. The two web-based prediction algorithms both gave long lists of genes, but shared relatively few genes. In addition, we searched in data obtained from the TCGA portal for negative correlations between miR-143 and mRNAs. Without further research, it is not clear which list contains the true targets of miRNA-143.

The only gene present in all three lists of putative target genes was *FXYD3/MAT8*. *FXYD3* has one site in its 3′ UTR were hybridization of miR-143 is predicted. In several cancer types expression of *FXYD3* appears to be deregulated [21–27]. Strikingly, *FXYD3* is transcriptionally activated by 5-fluorouracil treatment in the colon cancer cell line H630 [28]. If miR-143 negatively regulates FXYD3 expression then it is expected that low expression of FXYD3 is associated with worse PFS. This is indeed what we observed with the IHC analysis.

FXYD3 is a member of the small FXYD protein family. Each member is a small trans-membrane protein that has the short signature motif PFXYD (Pro, Phe, X, Tyr, Asp). It was shown that the FXYD proteins associate with the Na-K-ATPase (also known as sodium-potassium pump), which maintains the Na+ and K+ gradients across the plasma membrane. The FXYD proteins modulate the transport properties of the Na-K-ATPase [29, 30]. The Na+ and K+ gradient is essential in preservation of cell volume and the membrane potential. In addition, the Na+ gradient provides the energy for the activity of secondary transporters that transport numerous solutes, including other ions, glucose, and amino acids [31]. With this in mind it is conceivable that FXYD3 indirectly affects (nucleoside or nucleobase) transporters that are involved in the uptake of fluoropyrimidines (or metabolites of fluoropyrimidines), and that variations in expression of FXYD3 might lead to altered transport and could manifest as differences in cytotoxicity or response.

In summary, in this study we observe a relationship between miR-143 expression and PFS. Further pre-clinical studies are now necessary for the validation of miR-143 as predictive marker of the response to fluoropyrimidine-based chemotherapy in mCRC patients. In addition, investigation of the target genes of miR-143, and in particular FXYD3, will be useful to elucidate how fluoropyrimidines and miR-143 are connected.

## MATERIALS AND METHODS

### Patient selection

The patients included in this study participated in the CAIRO study (ClinTrials.gov NCT00312000) of the Dutch Colorectal Cancer Group (DCCG) [3]. In this multicenter phase III study, 820 patients with advanced CRC were randomized between sequential (Arm A) and combination (Arm B) treatment with capecitabine, irinotecan and oxaliplatin. The primary end point of the study was overall survival (OS), and was calculated as the interval from the date of randomization until death from any cause or until the date of last follow-up. The secondary objectives included PFS and tumor response. PFS for first-line treatment was calculated from the date of randomization to the first observation of disease progression or death from any cause. Assessment of tumor response was performed with computed tomography (CT) scans using to Response Evaluation Criteria for Solid Tumors (RECIST) criteria. The written informed consent required for all patients before study entry also included translational research on tumor tissue.

Immunohistochemistry analysis was performed on 243 tumor samples of patients treated with first line capecitabine monotherapy (arm A) for whom sufficient tumor material was available. For the miRNA analysis 55 patients treated with first line capecitabine monotherapy (arm A) were selected that had performance score 0, normal serum LDH, received at least 3 cycles of capecitabine, received no prior adjuvant chemotherapy and for whom formalin-fixed paraffin-embedded (FFPE) material of the primary tumor as well as normal tissue was available. Supplemental Table 1, 2 contain the baseline characteristics of the patients included in the protein and miRNA detection analyses, respectively.

### Immunohistochemistry on tissue microarrays

IHC analysis was performed on Tissue Micro Arrays (TMAs) containing primary tumor material. To assemble the arrays punches of 2 mm were taken from the FFPE primary tumor tissues. From each TMA, a 4 μm section was mounted on glass, de-paraffinised and re-hydrated.

The staining of Dicer was performed on a Ventana Benchmark XT autostainer with the XT ultraView DAB Kit (Ventana Medical Systems, Illkirch, France) using an antibody reacting with Dicer (Anti-DICER1, 1:75, Sigma–Aldrich, Hamburg, Germany). The slides were counterstained with Haematoxylin (Vector Laboratories). Different staining intensities were observed, and the scoring was performed as previously described [15]. Tumors were scored from 0 to 3, considering only the cytoplasmic area. Sections were evaluated three times, blinded to outcome data. The mean of all three values was then calculated and each sample was assigned to one of the four different IHC-staining categories (0–3).

For the staining of FXYD3, microwave antigen retrieval was performed using 10mM sodium citrate buffer (pH 6.0) for 10 minutes. After blocking endogenous peroxidase activity with 3% H_2_O_2_ for 20 minutes, slides were incubated with rabbit anti-human polyclonal FXYD3 antibody (Sigma-Aldrich, St. Louis, MO, USA, 1:200) overnight at 4°C. Subsequently, slides were incubated with Powervision Poly-HRP anti-Ms/Rb/Ra IgG (Immunologic, Duiven, The Netherlands) and developed using PowerDAB (Immunologic, Duiven, The Netherlands). Furthermore, slides were counterstained with hematoxylin (Vector Laboratories).

The staining intensity of the cytoplasm was graded as weak (light brown) and strong (brown). All sections were evaluated blinded to outcome data by two independent observers. Discrepancies in scoring were evaluated by the two observers together to obtain an agreement.

### RNA extraction and miRNA assays

Total RNA was isolated from FFPE tissue using the RecoverAllTM Total Nucleic Acid Isolation Kit (Applied Biosystems, Foster city, USA). To ensure a high percentage of tumor cells, the tumor tissue sections were macro dissected. Matched normal mucosal RNA was obtained from the resection margins or at least 1 cm distance from the tumor. In brief, four tissue sections of 20 μm were incubated with 100% xylene at 50°C to remove paraffin excess, followed by ethanol washes. Proteins were degraded by protease at 50° and 80°C. The RNA was extracted followed by nuclease digestion. Total RNA quantity and quality were determined using the Nanodrop 26 ND-1000 spectrophotometer (Nanodrop Technologies Inc., Wilmington, USA).

The expression levels of miRNAs were determined by means of Taqman microRNA assays (Supplemental Table 4 contains a list with the assays), following the manufacture's protocol (Applied Biosystems, Foster City, USA). First, cDNA was synthesized in triplicate from total RNA using the Human pool A Megaplex RT primers. Reverse transcriptase reactions were conducted using 66 ng total RNA, 2.67 mM dNTPs, 75 U MultiScribe Reverse Transcriptase, 1x RT buffer, 2 U RNase inhibitor, 3 mM MgCl2 and 1x Taqman MicroRNA RT Primers (Applied Biosystems, Foster city, USA). The 7.5 μl reactions were incubated for 40 cycles for 2 minutes at 16°C, 1 minute at 42°C and 1 second at 50°C followed by 5 minutes at 85°C for 5 minutes.

Pre-amplification was subsequently performed on 2.5 μl of synthesized cDNA in a total reaction of 22.5 μl of 1x Taqman PreAmp Master Mix (Applied Biosystems, Foster city, USA). The reactions were incubated for 10 minutes at 95°C, 2 minutes at 55°C, 2 minutes at 72°C, 12 cycles of 15 second at 95°C and 4 minutes at 60°C, 10 minutes at 99.9°C.

The quantitative PCR was performed in a total mixture of 10 μl consisting of 0.1 μl RT product (1:4 diluted from pre-amplified RT reaction), 1 x Taqman Universal PCR Master Mix (No AmpErase® UNG, Applied Biosystems, Foster City, USA) and 1 x the dedicated primer and probe mix. The reactions were incubated in a 96-well optical plate at 95°C for 10 minutes, followed by 40 cycles at 95°C for 15 seconds and at 60°C for 1 minute. All reactions were carried out in duplicate in a 7500 Real Time PCR System (Applied Biosystems, Foster City, USA). Due to the plate set-up it was necessary to correct for inter plate variation by incorporating an IPC, in triplicate (data for stability testing not shown). The threshold cycle (Cq) was defined as the fractional cycle number at which the fluorescence passes the fixed threshold. Relative quantification of miRNA expression was calculated using the ΔCq method using GenEx software [32].

### MiRNA target prediction

Lists of potential targets for miRNAs were created using the prediction algorithms TargetScan Release 6.2 and MicroCosm Targets Version 5 [33, 34].

To identify genes that show loss of expression upon increased miRNA expression, data from the Cancer Genome Atlas (TCGA) was downloaded on the 25^th^ of April 2013. The following filter settings were used to search in the data portal: Disease: COAD; Data Level: 3; Availability: Available. In total, we obtained IlluminaGA miRNASeq and RNASeq data for 177 tumor samples. The Spearman correlation and accompanying *p*-value was calculated for miR-143 expression and the expression of each mRNA. Genes with a negative rho and a *p*-value below 0.05 were considered putative target genes.

Overlaps between the lists of putative target genes were identified and visualized with a Venn diagram. Gene ontology analysis was performed using the web-based tool DAVID [35, 36].

### Data analysis

The qPCR data was analyzed using the GenEx software (MultID v.5.3.4, Göteborg, Sweden). Application of NormFinder resulted in the use of miR-17, miR-19a, miR-20a and miR-24 as reference genes, as its combined use led to the lowest accumulated standard deviation. The suitability of these genes was confirmed by GeNorm analysis [37, 38]. The miRNA expression in tumor tissue is compared with the expression in normal tissue using the Wilcoxon signed rank test. The Kaplan-Meier method was used to make survival curves. The survival distributions were compared with the log-rank test. The distributions of clinico-pathological characteristics in different groups were compared with Fisher's exact test or Wilcoxon rank sum test. Correction for multiple testing was performed with the Benjamini & Hochberg adjustment method. Multivariate survival analysis was performed with Cox proportional hazards regression. Statistical procedures were performed with R.

## SUPPLEMENTARY MATERIAL TABLES


