# Supplementary material for: MicroRNA-143 is a putative predictive factor for the response to fluoropyrimidine-based chemotherapy in patients with metastatic colorectal cancer
Source: Oncotarget. 2015 May 8;6(26):22996–3007. doi: 10.18632/oncotarget.4035 (PMC4673216; doi:10.18632/oncotarget.4035)
Supplement: Supplementary file 1 [file oncotarget-06-22996-s001.pdf]

MicroRNA-143 is a putative predictive factor for the response to fluoropyrimidine-based chemotherapy in patients with metastatic colorectal cancer

Supplementary Material

Supplemental Table 1: characteristics of patients and tumors in the Dicer IHC experiment

Feature		All (n=243)	%
**Gender**			
	Male	155	64%
	Female	88	36%
**Age**			
	Median (range)	64 (36-83)	-
**Performance status**			
	PS0	166	68%
	PS1	67	28%
	PS2	10	4%
**LDH serum**			
	Normal	169	70%
	Abnormal	74	30%
**Previous adjuvant therapy**			
	Yes	39	16%
	No	204	84%
**Number of cycles of Capecitabine**			
	Median (range)	7 (1-53)	-
**Diagnosis of metastasis (12 months)**			
	Synchronous	139	57%
	Metachronous	104	43%
**Predominant localization of metastases**			
	Liver	170	70%
	Extra-hepatic	72	30%
	Unknown	1	0%
**Metastatic sites involved**			

1	113	47%
≥ 2	129	53%
Unknown	1	0%
**Localization of the primary tumor**		
Colon	161	66%
Rectosigmoid	13	5%
Rectum	67	28%
Multiple tumor	2	1%
**Histology of the primary tumor**		
Adenocarcinoma	204	84%
Mucinous adenocarcinoma (>50% WHO)	17	7%
Other	2	1%
Unknown	20	8%
**Differentiation grade**		
Good	10	4%
Moderate	112	46%
Poor	100	41%
Undifferentiated	2	1%
Unknown	19	8%
**T stage**		
T1	1	0%
T2	14	6%
T3	181	74%
T4	42	17%
Unknown	5	2%
**N stage**		
N0	71	29%
N1	86	35%
N2	79	33%
Unknown	7	3%
**MSI status**		
dMMR	4	2%
pMMR	212	87%
Unknown	27	11%

Supplemental Table 2: characteristics of patients and tumors in the miRNA expression analysis

Feature		miR-143 expression in primary tumor			Statistical test	p-Value
		All (n=55)	Low/1 (n=27)	High/2 (n=28)		
**Gender**					Fisher's Exact	0.562
	Male	38	20	18		
	Female	17	7	10		
**Age**					Kruskal-Wallis	0.007
	Median (range)	65 (34-79)	69 (45-79)	64 (34-75)		
**Performance status**					n.d.	n.d.
	PS0	55	27	28		
	PS1	0	0	0		
	PS2	0	0	0		
**LDH serum**					n.d.	n.d.
	Normal	55	27	28		
	Abnormal	0	0	0		
**Previous adjuvant therapy**					n.d.	n.d.
	Yes	0	0	0		
	No	55	27	28		
**Number of cycles of Capecitabine**					Kruskal-Wallis	0.012
	Median (range)	9 (3-32)	9 (3-32)	6 (3-32)		
**Diagnosis of metastasis (12 months)**					Fisher's Exact	0.588
	Synchronous	32	17	15		
	Metachronous	23	10	13		
**Predominant localization of metastases**					Fisher's Exact	1.000
	Liver	38	19	19		
	Extra-hepatic	15	7	8		
	Unknown	2	1	1		
**Metastatic sites involved**					Fisher's Exact	0.588
	1	23	10	13		
	≥ 2	32	17	15		
	Unknown	0	0	0		
**Localization of the primary tumor**					Fisher's Exact	0.469
	Colon	47	22	25		

Rectosigmoid	8	5	3		
Rectum	0	0	0		
Multiple tumor	0	0	0		
**Histology of the primary tumor**				Fisher's Exact	1.000
Adenocarcinoma	41	22	22		
Mucinous adenocarcinoma (>50% WHO)	7	3	4		
Other	0	0	0		
Unknown	4	2	2		
**Differentiation grade**				Fisher's Exact	0.882
Good	1	1	0		
Moderate	32	15	17		
Poor	18	9	9		
Undifferentiated	0	0	0		
Unknown	4	2	2		
**T stage**				Fisher's Exact	0.866
T1	0	0	0		
T2	2	1	1		
T3	40	18	22		
T4	11	6	5		
Unknown	2	2	0		
**N stage**				Fisher's Exact	0.279
N0	24	11	13		
N1	19	7	12		
N2	10	7	3		
Unknown	2	2	0		
**MSI status**				Fisher's Exact	1.000
dMMR	2	1	1		
pMMR	46	21	25		
Unknown	7	5	2		

n.d. = not done

Supplemental Table 3: characteristics of patients and tumors in the FXYD3 IHC experiment

Feature		FXYD3 staining in primary tumor		Statistical test	p-Value
		Weak/1 (n=32)	Strong/2 (n=211)		
**Gender**				Fisher's Exact	0.236
	Male	17	138		
	Female	15	73		
**Age**				Kruskal-Wallis	0.677
	Median (range)	65 (40-77)	64 (36-83)		
**Performance status**				Fisher's Exact	0.774
	PS0	22	144		
	PS1	8	59		
	PS2	2	8		
**LDH serum**				Fisher's Exact	0.0988
	Normal	18	151		
	Abnormal	14	60		
**Previous adjuvant therapy**				Fisher's Exact	0.1921
	Yes	8	31		
	No	24	180		
**Number of cycles of Capecitabine**				Kruskal-Wallis	0.169
	Median (range)	6 (1-27)	7 (1-53)		
**Diagnosis of metastasis (12 months)**				Fisher's Exact	0.702
	Synchronous	17	122		
	Metachronous	15	89		
**Predominant localization of metastases**				Fisher's Exact	0.095
	Liver	18	152		
	Extra-hepatic	14	58		
	Unknown	0	1		
**Metastatic sites involved**				Fisher's Exact	0.708
	1	16	97		
	≥ 2	16	113		
	Unknown	0	1		
**Localization of the primary tumor**				Fisher's Exact	0.504

Colon	24	137		
Rectosigmoid	0	13		
Rectum	8	59		
Multiple tumor	0	2		
**Histology of the primary tumor**			Fisher's Exact	0.021
Adenocarcinoma	25	179		
Mucinous adenocarcinoma (>50% WHO)	2	15		
Other	2	0		
Unknown	3	17		
**Differentiation grade**			Fisher's Exact	0.024
Good	0	10		
Moderate	13	99		
Poor	14	86		
Undifferentiated	2	0		
Unknown	3	16		
**T stage**			Fisher's Exact	0.193
T1	0	1		
T2	4	10		
T3	20	161		
T4	7	35		
Unknown	1	4		
**N stage**			Fisher's Exact	0.089
N0	11	60		
N1	6	80		
N2	14	65		
Unknown	1	6		
**MSI status**			Fisher's Exact	1.000
dMMR	0	4		
pMMR	29	183		
Unknown	3	24		

Supplemental Table 4: Taqman microRNA assays

Assay Name	Assay ID	Target Sequence	Reference
miR-16	391	UAGCAGCACGUAAAUAUUGGCG	[1]
miR-17	2308	CAAAGUGCUUACAGUGCAGGUAG	[2]
miR-18a	2422	UAAGGUGCAUCUAGUGCAGAUAG	[2]
miR-19a	395	UGUGCAAAUCUAUGCAAAACUGA	[2]
miR-19b	396	UGUGCAAAUCCAUGCAAAACUGA	[2]
miR-20a	580	UAAAGUGCUUAUAGUGCAGGUAG	[2]; [3]
miR-21	397	UAGCUUAUCAGACUGAUGUUGA	[4]; [5]; [6]
miR-24	402	UGGCUCAGUUCAGCAGGAACAG	[7]; [8]
miR-26b	407	UUCAAGUAAUUCAGGAUAGGU	[9]; [10]
miR-31	2279	AGGCAAGAUGCUGGCAUAGCU	[11]
MiR-34a	426	UGGCAGUGUCUUAGCUGGUUGU	[12]
miR-92a	431	UAUUGCACUUGUCCCGGCCUGU	[13]; [14]
MiR-93	1090	CAAAGUGCUGUUCGUGCAGGUAG	[14]
miR-103	439	AGCAGCAUUGUACAGGGCUAUGA	[15]; [16]
MiR-125b	449	UCCCUGAGACCCUAACUUGUGA	[17]

MiR-137	1129	UUAUUGCUUAAGAAUACGCGUAG	[17]
MiR-140	1187	CAGUGGUUUUACCCUAUGGUAG	[18]
MiR-143	2249	UGAGAUGAAGCACUGUAGCUC	[19]
MiR-145	2278	GUCCAGUUUUCCCAGGAAUCCCU	[20]
MiR-148a	470	UCAGUGCACUACAGAACUUUGU	[21]; [22]
MiR-191	2299	CAACGGAAUCCCAAAAGCAGCUG	[14]
MiR-192	491	CUGACCUAUGAAUUGACAGCC	[23]
MiR-215	518	AUGACCUAUGAAUUGACAGAC	[23]
MiR-222	2276	AGCUACAUCUGGCUACUGGGU	[14]
let-7a	377	UGAGGUAGUAGGUUGUAUAGUU	[24, 25]
let-7g	2282	UGAGGUAGUAGUUUGUACAGUU	[26]

References for Supplemental Table 4

1. Xia L, Zhang D, Du R, Pan Y, Zhao L, Sun S, Hong L, Liu J and Fan D. miR-15b and miR-16 modulate multidrug resistance by targeting BCL2 in human gastric cancer cells. International journal of cancer Journal international du cancer. 2008; 123(2):372-379.
2. Zhao HY, Ooyama A, Yamamoto M, Ikeda R, Haraguchi M, Tabata S, Furukawa T, Che XF, Iwashita K, Oka T, Fukushima M, Nakagawa M, Ono M, Kuwano M and Akiyama S. Down regulation of c-Myc and induction of an angiogenesis inhibitor, thrombospondin-1, by 5-FU in human colon cancer KM12C cells. Cancer letters. 2008; 270(1):156-163.
3. Chai H, Liu M, Tian R, Li X and Tang H. miR-20a targets BNIP2 and contributes chemotherapeutic resistance in colorectal adenocarcinoma SW480 and SW620 cell lines. Acta biochimica et biophysica Sinica. 2011; 43(3):217-225.
4. Schetter AJ, Leung SY, Sohn JJ, Zanetti KA, Bowman ED, Yanaihara N, Yuen ST, Chan TL, Kwong DL, Au GK, Liu CG, Calin GA, Croce CM and Harris CC. MicroRNA expression profiles associated with prognosis and therapeutic outcome in colon adenocarcinoma. JAMA : the journal of the American Medical Association. 2008; 299(4):425-436.

5. Valeri N, Gasparini P, Braconi C, Paone A, Lovat F, Fabbri M, Sumani KM, Alder H, Amadori D, Patel T, Nuovo GJ, Fishel R and Croce CM. MicroRNA-21 induces resistance to 5-fluorouracil by down-regulating human DNA MutS homolog 2 (hMSH2). Proceedings of the National Academy of Sciences of the United States of America. 2010; 107(49):21098-21103.
6. Tomimaru Y, Eguchi H, Nagano H, Wada H, Tomokuni A, Kobayashi S, Marubashi S, Takeda Y, Tanemura M, Umeshita K, Doki Y and Mori M. MicroRNA-21 induces resistance to the anti-tumour effect of interferon-alpha/5-fluorouracil in hepatocellular carcinoma cells. British journal of cancer. 2010; 103(10):1617-1626.
7. Gmeiner WH, Reinhold WC and Pommier Y. Genome-wide mRNA and microRNA profiling of the NCI 60 cell-line screen and comparison of FdUMP[10] with fluorouracil, floxuridine, and topoisomerase 1 poisons. Molecular cancer therapeutics. 2010; 9(12):3105-3114.
8. Mishra PJ, Song B, Mishra PJ, Wang Y, Humeniuk R, Banerjee D, Merlino G, Ju J and Bertino JR. MiR-24 tumor suppressor activity is regulated independent of p53 and through a target site polymorphism. PloS one. 2009; 4(12):e8445.
9. Ma YL, Zhang P, Wang F, Moyer MP, Yang JJ, Liu ZH, Peng JY, Chen HQ, Zhou YK, Liu WJ and Qin HL. Human embryonic stem cells and metastatic colorectal cancer cells shared the common endogenous human microRNA-26b. Journal of cellular and molecular medicine. 2011; 15(9):1941-1954.
10. Ji J, Shi J, Budhu A, Yu Z, Forgues M, Roessler S, Ambs S, Chen Y, Meltzer PS, Croce CM, Qin LX, Man K, Lo CM, Lee J, Ng IO, Fan J, et al. MicroRNA expression, survival, and response to interferon in liver cancer. The New England journal of medicine. 2009; 361(15):1437-1447.
11. Wang CJ, Stratmann J, Zhou ZG and Sun XF. Suppression of microRNA-31 increases sensitivity to 5-FU at an early stage, and affects cell migration and invasion in HCT-116 colon cancer cells. BMC cancer. 2010; 10:616.
12. Akao Y, Noguchi S, Iio A, Kojima K, Takagi T and Naoe T. Dysregulation of microRNA-34a expression causes drug-resistance to 5-FU in human colon cancer DLD-1 cells. Cancer letters. 2011; 300(2):197-204.
13. Tsuchida A, Ohno S, Wu W, Borjigin N, Fujita K, Aoki T, Ueda S, Takanashi M and Kuroda M. miR-92 is a key oncogenic component of the miR-17-92 cluster in colon cancer. Cancer science. 2011; 102(12):2264-2271.
14. Zhou J, Zhou Y, Yin B, Hao W, Zhao L, Ju W and Bai C. 5-Fluorouracil and oxaliplatin modify the expression profiles of microRNAs in human colon cancer cells in vitro. Oncology reports. 2010; 23(1):121-128.
15. Chen HY, Lin YM, Chung HC, Lang YD, Lin CJ, Huang J, Wang WC, Lin FM, Chen Z, Huang HD, Shyy JY, Liang JT and Chen RH. miR-103/107 promote metastasis of colorectal cancer by targeting the metastasis suppressors DAPK and KLF4. Cancer research. 2012; 72(14):3631-3641.
16. Ren C, Chen H, Han C, Wang D and Fu D. Increased plasma microRNA and CD133/CK18-positive cancer cells in the pleural fluid of a pancreatic cancer patient with liver and pleural metastases and correlation with chemoresistance. Oncology letters. 2012; 4(4):691-694.
17. Svoboda M, Izakovicova Holla L, Sefr R, Vrtkova I, Kocakova I, Tichy B and Dvorak J. Micro-RNAs miR125b and miR137 are frequently upregulated in response to capecitabine chemoradiotherapy of rectal cancer. International journal of oncology. 2008; 33(3):541-547.
18. Song B, Wang Y, Xi Y, Kudo K, Bruheim S, Botchkina GI, Gavin E, Wan Y, Formentini A, Kornmann M, Fodstad O and Ju J. Mechanism of chemoresistance mediated by miR-140 in human osteosarcoma and colon cancer cells. Oncogene. 2009; 28(46):4065-4074.
19. Borralho PM, Kren BT, Castro RE, da Silva IB, Steer CJ and Rodrigues CM. MicroRNA-143 reduces viability and increases sensitivity to 5-fluorouracil in HCT116 human colorectal cancer cells. The FEBS journal. 2009; 276(22):6689-6700.
20. Takagi T, Iio A, Nakagawa Y, Naoe T, Tanigawa N and Akao Y. Decreased expression of microRNA-143 and -145 in human gastric cancers. Oncology. 2009; 77(1):12-21.

21. Takahashi M, Cuatrecasas M, Balaguer F, Hur K, Toiyama Y, Castells A, Boland CR and Goel A. The clinical significance of MiR-148a as a predictive biomarker in patients with advanced colorectal cancer. PloS one. 2012; 7(10):e46684.
22. Hummel R, Watson DI, Smith C, Kist J, Michael MZ, Haier J and Hussey DJ. Mir-148a improves response to chemotherapy in sensitive and resistant oesophageal adenocarcinoma and squamous cell carcinoma cells. Journal of gastrointestinal surgery : official journal of the Society for Surgery of the Alimentary Tract. 2011; 15(3):429-438.
23. Boni V, Bitarte N, Cristobal I, Zarate R, Rodriguez J, Maiello E, Garcia-Foncillas J and Bandres E. miR-192/miR-215 influence 5-fluorouracil resistance through cell cycle-mediated mechanisms complementary to its post-transcriptional thymidilate synthase regulation. Molecular cancer therapeutics. 2010; 9(8):2265-2275.
24. Tsang WP and Kwok TT. Let-7a microRNA suppresses therapeutics-induced cancer cell death by targeting caspase-3. Apoptosis : an international journal on programmed cell death. 2008; 13(10):1215-1222.
25. Kjersem JB, Ikdahl T, Guren T, Skovlund E, Sorbye H, Hamfjord J, Pfeiffer P, Glimelius B, Kersten C, Solvang H, Tveit KM and Kure EH. Let-7 miRNA-binding site polymorphism in the KRAS 3'UTR; colorectal cancer screening population prevalence and influence on clinical outcome in patients with metastatic colorectal cancer treated with 5-fluorouracil and oxaliplatin +/- cetuximab. BMC cancer. 2012; 12:534.
26. Nakajima G, Hayashi K, Xi Y, Kudo K, Uchida K, Takasaki K, Yamamoto M and Ju J. Non-coding MicroRNAs hsa-let-7g and hsa-miR-181b are Associated with Chemoresponse to S-1 in Colon Cancer. Cancer genomics & proteomics. 2006; 3(5):317-324.

Supplemental Table 5: putative target genes for miR-143 identified with Microcosm

#	GENE SYMBOL	TRANSCRIPT_ID	SCORE	PVALUE_OG
1	SPINT3	ENST00000217428	203,468	7.25E+02
2	K0256	ENST00000380927	194,323	4.02E+00
3	C7orf34	ENST00000297830	194,323	1.85E+03
4	TMEM16G	ENST00000274979	191,351	5.78E+02
5	CAB39L	ENST00000347776	190,894	8.95E+01
6	GRIK3	ENST00000373091	189,835	2.92E+03
7	C10orf99	ENST00000372126	187,897	2.83E+01
8	HERC5	ENST00000264350	186,322	4.18E+03
9	GRIPAP1	ENST00000015926	186,074	3.15E+03
10	ADAMTSL1	ENST00000327883	185,179	4.70E+03
11	EME1	ENST00000338165	185,056	5.67E+02
12	BRD2	ENST00000374831	184,548	5.01E+03
13	FMR1NB	ENST00000370465	184,036	8.95E+01
14	IFNWP18	ENST00000239347	184,036	5.28E+03
15	KIF9	ENST00000335044	184,036	5.28E+03
16	EMID1	ENST00000334018	183,845	3.65E+03
17	Q6H1K9	ENST00000376242	183,812	1.38E+02
18	NXF1	ENST00000294172	183,757	5.43E+03
19	GNAS	ENST00000371081	181,974	6.51E+03
20	NP_071353.4	ENST00000184956	181,856	5.47E+02
21	ECSIT	ENST00000270517	181,749	3.94E+01
22	HMBS	ENST00000278715	181,749	7.41E+01
23	PLA2G1B	ENST00000308366	181,749	2.10E+03
24	DEFA6	ENST00000382702	181,749	5.73E+03
25	DEFA6	ENST00000297436	181,749	6.66E+03
26	C7orf28B	ENST00000316731	181,749	6.66E+03
27	C7orf28A	ENST00000325974	181,749	6.66E+03
28	SMNDC1	ENST00000369592	181,749	6.66E+03
29	MAPK13	ENST00000373766	181,109	7.11E+03
30	ADFP	ENST00000276914	180,917	5.35E+02
31	MAPK7	ENST00000299612	180,606	1.51E+00

32	CIDEA	ENST00000342845	180,606	1.08E+03
33	MRO	ENST00000256425	180,606	3.61E+03
34	NP_079189.3	ENST00000340646	180,606	7.48E+03
35	DDC	ENST00000380984	180,606	7.48E+03
36	TIGD5	ENST00000321385	180,364	1.82E+03
37	SPINT3	ENST00000372673	180,011	7.94E+03
38	C1orf49	ENST00000319416	179,463	3.45E+01
39	ZNF570	ENST00000330173	179,463	1.11E+03
40	TMEM60	ENST00000257663	179,463	1.31E+03
41	UBE2T	ENST00000367274	179,463	6.48E+03
42	TFF3	ENST00000291525	179,463	8.40E+03
43	BAG3	ENST00000369085	178,983	1.90E+03
44	CIR	ENST00000342016	178,892	8.90E+03
45	EIF4ENIF1	ENST00000330125	178,862	7.83E+03
46	BCAP31	ENST00000345046	178,596	9.17E+03
47	GNAS	ENST00000371099	178,234	9.51E+03
48	PLA1A	ENST00000273371	178,149	2.30E+02
49	Q5JXA8	ENST00000381630	178,068	9.68E+03
50	SLC17A1	ENST00000377886	177,898	9.84E+03
51	TMEM121	ENST00000330164	177,787	1.72E+00
52	KLK8	ENST00000376832	177,706	1.00E+04
53	GZMK	ENST00000381400	177,666	1.01E+04
54	WDR69	ENST00000309931	177,335	1.04E+04
55	PDLIM5	ENST00000359265	177,233	1.05E+04
56	SNIP	ENST00000264659	177,177	8.62E+02
57	USMG5P1	ENST00000373490	177,177	1.06E+04
58	SMC2	ENST00000374787	177,168	3.49E+02
59	AADAC	ENST00000232892	176,777	5.16E+02
60	CLRN3	ENST00000368671	176,646	5.64E+03
61	Q8N793	ENST00000359068	176,342	1.15E+04
62	Q8N793	ENST00000355435	176,306	1.16E+04
63	Q6ZU57	ENST00000360524	176,297	1.16E+04
64	PRPF19	ENST00000227524	176,208	2.46E+01

65	WNK4	ENST00000246914	176,075	7.06E+03
66	GCS1	ENST00000233616	176,034	1.19E+02
67	IAG2	ENST00000373338	176,034	7.06E+02
68	C5orf14	ENST00000358387	176,034	1.29E+03
69	SEC61G	ENST00000352861	176,034	8.32E+03
70	RQCD1	ENST00000295701	176,034	1.19E+04
71	DEFA3	ENST00000327857	176,034	1.19E+04
72	KRT28	ENST00000377794	176,034	1.19E+04
73	DEFA3	ENST00000382679	176,034	1.19E+04
74	AMICA1	ENST00000356289	175,854	1.21E+04
75	AMICA1	ENST00000292067	175,691	1.23E+04
76	RPS27	ENST00000368565	175,398	1.27E+04
77	CCDC94	ENST00000262962	175,348	6.04E+00
78	CAB39L	ENST00000347776	174,891	8.95E+01
79	FMR1NB	ENST00000370465	174,891	8.95E+01
80	KIF9	ENST00000265529	174,891	2.18E+02
81	SCN8A	ENST00000357961	174,891	1.33E+04
82	EDF1	ENST00000371649	174,853	1.34E+04
83	HLA-DRB5	ENST00000374975	174,793	1.35E+04
84	RNF170	ENST00000330830	174,791	4.36E+02
85	IFNWP18	ENST00000259555	174,758	1.39E+03
86	UBE2E4P	ENST00000305934	174,696	8.32E+03
87	SFRS11	ENST00000370951	174,466	1.39E+04
88	IFNWP18	ENST00000380220	174,431	1.40E+04
89	IFNG	ENST00000229135	174,201	3.83E+03
90	LHX2	ENST00000373615	174,193	1.54E+03
91	DHX8	ENST00000262415	174,167	6.44E+03
92	NP_001010859.1	ENST00000382097	174,081	1.45E+04
93	PTPRD	ENST00000356435	173,887	9.64E+03
94	TMEM126B	ENST00000358867	173,748	3.45E+01
95	ABHD14A	ENST00000360889	173,748	3.34E+02
96	SFRS16	ENST00000221455	173,748	3.32E+03
97	HEXIM2	ENST00000307275	173,748	5.94E+03

98	TUBG2	ENST00000251412	173,748	1.12E+04
99	CSH2	ENST00000345366	173,748	1.50E+04
100	C9orf163	ENST00000354376	173,725	1.50E+04
101	IFNA16	ENST00000380216	173,672	1.51E+04
102	RFPL3	ENST00000382088	173,672	1.51E+04
103	ANKRD46	ENST00000358990	173,309	1.11E+02
104	ITGB3BP	ENST00000283568	172,999	1.62E+04
105	KIAA0372	ENST00000380021	172,996	1.19E+04
106	ZCCHC7	ENST00000322831	172,888	1.63E+04
107	C7orf38	ENST00000333062	172,873	1.64E+04
108	SORCS1	ENST00000369698	172,771	1.65E+04
109	Q6H1K9	ENST00000376242	172,739	1.38E+02
110	IL1F9	ENST00000259205	172,612	8.40E+03
111	CCDC58	ENST00000291458	172,605	1.05E+02
112	BPIL1	ENST00000170150	172,605	4.17E+03
113	NPPA	ENST00000376480	172,605	6.84E+03
114	NUP88	ENST00000381211	172,605	8.25E+03
115	PTPN23	ENST00000265562	172,605	1.68E+04
116	C1orf53	ENST00000367393	172,605	1.68E+04
117	NPPA	ENST00000376476	172,605	1.68E+04
118	PC	ENST00000355677	172,367	4.51E+01
119	ZMYND19	ENST00000298585	172,357	5.45E+03
120	ASXL1	ENST00000375689	172,315	2.13E+03
121	OR7A17	ENST00000327462	172,169	1.76E+04
122	PARP6	ENST00000336471	172,116	1.77E+04
123	LENEP	ENST00000368427	171,936	1.52E+04
124	RASEF	ENST00000340717	171,921	1.80E+04
125	VHL	ENST00000256474	171,865	1.81E+04
126	MFSD11	ENST00000336509	171,861	1.11E+04
127	CLDN10	ENST00000376855	171,751	1.83E+04
128	CTRC	ENST00000375943	171,554	1.87E+04
129	KIF4A	ENST00000374403	171,553	1.87E+04
130	CCBL1	ENST00000302586	171,471	3.67E+03

131	ZUBR1	ENST00000375224	171,463	1.89E+04
132	ATP12A	ENST00000218548	171,462	2.71E+02
133	TMEM134	ENST00000308022	171,462	1.80E+03
134	ARHGAP15	ENST00000295095	171,462	2.08E+03
135	NP_955369.1	ENST00000333449	171,462	4.49E+03
136	SCEL	ENST00000349847	171,462	8.22E+03
137	CRYM	ENST00000219599	171,462	1.89E+04
138	GNL3	ENST00000354540	171,462	1.89E+04
139	ENSA	ENST00000362052	171,462	1.89E+04
140	ARPC5	ENST00000367534	171,462	1.89E+04
141	PRPS1	ENST00000372418	171,462	1.89E+04
142	SCEL	ENST00000377246	171,462	1.89E+04
143	SH3BGR	ENST00000380631	171,462	1.89E+04
144	SH3BGR	ENST00000380634	171,462	1.89E+04
145	ATP12A	ENST00000381946	171,462	1.89E+04
146	C8orf45	ENST00000313934	171,266	1.92E+04
147	CD79A	ENST00000221972	171,006	1.91E+04
148	C20orf112	ENST00000375675	171,005	1.97E+04
149	TXNDC6	ENST00000341790	170,815	2.01E+04
150	LMO4	ENST00000370542	170,811	2.01E+04
151	HOXA5	ENST00000222726	170,795	1.04E+03
152	Q96HM8	ENST00000355278	170,772	2.02E+04
153	NP_001067.2	ENST00000338206	170,654	2.04E+04
154	NM_203423	ENST00000318930	170,572	1.05E+03
155	NM_203423	ENST00000382541	170,511	1.06E+03
156	NP_955373.2	ENST00000328759	170,474	2.03E+04
157	Q5JPQ1	ENST00000370707	170,455	2.09E+04
158	LAD1	ENST00000367313	170,358	8.22E+03
159	C15orf23	ENST00000249776	170,319	2.05E+03
160	C6orf15	ENST00000259870	170,319	2.11E+04
161	GEMIN8	ENST00000332885	170,319	2.11E+04
162	GABBR1	ENST00000376998	170,319	2.11E+04
163	MLLT10	ENST00000377100	170,319	2.11E+04

164	RB1	ENST00000378437	170,319	2.11E+04
165	GEMIN8	ENST00000380523	170,319	2.11E+04
166	STIP1	ENST00000358794	170,242	2.13E+04
167	MYO3A	ENST00000265944	170,172	1.11E+03
168	PIK3CA	ENST00000263967	170,146	2.12E+03
169	DTNB	ENST00000288642	170,146	2.15E+04
170	PRUNE2	ENST00000376710	170,114	2.16E+04
171	STIP1	ENST00000305218	170,103	2.16E+04
172	HS2ST1	ENST00000370551	170,046	2.17E+04
173	C14orf8	ENST00000321760	169,999	7.60E+02
174	TINAG	ENST00000370864	169,982	2.19E+04
175	FRY	ENST00000380235	169,965	2.19E+04
176	COX7A2L	ENST00000234301	169,844	2.22E+04
177	C1orf19	ENST00000367517	169,732	2.24E+04
178	FXYD3	ENST00000344013	169,403	1.97E+02
179	MLXIPL	ENST00000354613	169,395	2.32E+04
180	KLHL6	ENST00000341319	169,305	2.34E+04
181	PARC	ENST00000354351	169,185	2.37E+04
182	CDKL3	ENST00000265334	169,176	4.94E+02
183	UTP18	ENST00000225298	169,176	5.03E+03
184	MYL3	ENST00000292327	169,176	8.60E+03
185	TTC12	ENST00000314756	169,176	8.66E+03
186	NP_071395.1	ENST00000310686	169,176	2.37E+04
187	KIAA0748	ENST00000316577	169,176	2.37E+04
188	C18orf20	ENST00000323355	169,176	2.37E+04
189	PROZ	ENST00000342783	169,176	2.37E+04
190	STK16	ENST00000358905	169,176	2.37E+04
191	SYTL3	ENST00000360448	169,176	2.37E+04
192	MOSC2	ENST00000366914	169,176	2.37E+04
193	PRIM2A	ENST00000370687	169,176	2.37E+04
194	PROZ	ENST00000375547	169,176	2.37E+04
195	USE1	ENST00000379776	169,176	2.37E+04
196	PLAUR	ENST00000339082	169,156	2.38E+04

197	hCG_1790474	ENST00000361429	169,062	2.38E+04
198	DYRK1B	ENST00000323039	168,983	2.42E+04
199	HLA-DRB1	ENST00000360004	168,952	2.42E+04
200	OR7C1	ENST00000248073	168,806	2.46E+04
201	BAGE	ENST00000335369	168,806	2.46E+04
202	CD33	ENST00000262262	168,657	1.11E+04
203	Q96HG1	ENST00000330288	168,564	2.52E+04
204	KIR2DL1	ENST00000360233	168,468	2.54E+04
205	MYBL2	ENST00000217026	168,451	2.85E+03
206	ABCA4	ENST00000361520	168,357	3.12E+02
207	HVCN1	ENST00000356742	168,317	7.57E+03
208	ABCA4	ENST00000370225	168,216	2.61E+04
209	SMC2	ENST00000374787	168,141	3.49E+02
210	HIST1H1E	ENST00000377787	168,125	2.63E+04
211	TMEM126B	ENST00000358867	168,033	3.45E+01
212	KIAA0274	ENST00000230124	168,033	1.37E+02
213	Q6ZP14	ENST00000378347	168,033	8.85E+02
214	GRTP1	ENST00000375431	168,033	2.12E+03
215	SLC22A16	ENST00000368919	168,033	3.82E+03
216	TAL2	ENST00000374699	168,033	4.01E+03
217	MRPS24	ENST00000317534	168,033	5.75E+03
218	ALS2CR11	ENST00000286195	168,033	6.55E+03
219	GPR18	ENST00000340807	168,033	9.23E+03
220	DPM1	ENST00000371584	168,033	1.10E+04
221	SLC22A16	ENST00000330550	168,033	2.66E+04
222	ZNF394	ENST00000337673	168,033	2.66E+04
223	VPS72	ENST00000354473	168,033	2.66E+04
224	ZMYM2	ENST00000382881	168,033	2.66E+04
225	SFRS11	ENST00000370949	168,007	2.66E+04
226	SKIL	ENST00000259119	167,996	2.67E+04
227	MAGEA9B	ENST00000243314	167,981	2.67E+04
228	MAGEA9	ENST00000298974	167,981	2.67E+04
229	PRPF18	ENST00000298451	167,965	2.68E+04

230	M6PR	ENST00000000412	167,913	3.33E+03
231	TDO2	ENST00000281525	167,848	1.21E+02
232	KRT2	ENST00000309680	167,834	2.71E+04
233	CDC6	ENST00000209728	167,824	3.48E+01
234	RAB9A	ENST00000243325	167,808	2.72E+04
235	RABL4	ENST00000381855	167,801	3.53E+02
236	Q8NFD4	ENST00000377519	167,801	2.72E+04
237	ATP10A	ENST00000389967	167,801	2.72E+04
238	PRTG	ENST00000389286	167,732	1.23E+04
239	PSG2	ENST00000378039	167,639	2.76E+04
240	PLAU	ENST00000372762	167,599	2.77E+04
241	EAPP	ENST00000250454	167,448	9.62E+01
242	RMND1	ENST00000367299	167,423	2.82E+04
243	XR_017872.1	ENST00000331301	167,366	2.84E+04
244	NP_775898.2	ENST00000323854	167,357	2.84E+04
245	C1orf80	ENST00000334051	167,316	2.85E+04
246	SLC22A7	ENST00000372585	167,261	2.57E+03
247	CRELD1	ENST00000326434	167,185	2.89E+04
248	DLEU1	ENST00000378180	167,135	2.91E+04
249	C19orf10	ENST00000262947	167,104	1.55E+03
250	CDC20B	ENST00000331730	167,101	2.92E+04
251	FBXO16	ENST00000380254	167,101	2.92E+04
252	FGD6	ENST00000343958	167,097	2.92E+04
253	C6orf130	ENST00000244558	167,027	8.87E+03
254	TAOK2	ENST00000308893	166,998	1.53E+04
255	C6orf130	ENST00000373154	166,994	2.95E+04
256	IGLV1-44	ENST00000390297	166,948	2.96E+04
257	NP_060761.2	ENST00000371218	166,889	7.00E+01
258	CASP8	ENST00000343290	166,889	1.30E+02
259	CASP8	ENST00000343290	166,889	1.30E+02
260	ZNF277P	ENST00000361822	166,889	2.09E+02
261	NFATC2	ENST00000371567	166,889	3.66E+02
262	MRPS33	ENST00000324787	166,889	4.75E+02

263	RNF175	ENST00000274068	166,889	3.05E+03
264	SHC3	ENST00000375831	166,889	4.17E+03
265	FKBP3	ENST00000216330	166,889	4.40E+03
266	MOGAT3	ENST00000223114	166,889	2.98E+04
267	ACAT1	ENST00000265838	166,889	2.98E+04
268	S100A11	ENST00000271638	166,889	2.98E+04
269	XR_017721.1	ENST00000283760	166,889	2.98E+04
270	NFIL3	ENST00000297689	166,889	2.98E+04
271	NP_060761.2	ENST00000303721	166,889	2.98E+04
272	LOC653075	ENST00000326816	166,889	2.98E+04
273	HSD17B7	ENST00000334622	166,889	2.98E+04
274	LOC653720	ENST00000340249	166,889	2.98E+04
275	LOC653125	ENST00000342893	166,889	2.98E+04
276	XR_017846.1	ENST00000360934	166,889	2.98E+04
277	NP_060761.2	ENST00000371212	166,889	2.98E+04
278	NFIL3	ENST00000375724	166,826	2.58E+03
279	DOCK2	ENST00000343291	166,826	3.00E+04
280	CALN1	ENST00000329008	166,796	4.08E+03
281	O52L2	ENST00000316540	166,796	3.01E+04
282	Q96J93	ENST00000360562	166,796	3.01E+04
283	TXNDC8	ENST00000374507	166,796	3.01E+04
284	Q53S57	ENST00000377435	166,796	3.01E+04
285	UBAC2	ENST00000376429	166,687	3.04E+04
286	RPS24	ENST00000372355	166,682	3.04E+04
287	CLGN	ENST00000325617	166,542	1.31E+03
288	KRT76	ENST00000332411	166,541	3.08E+04
289	SBDS	ENST00000246868	166,513	8.61E+03
290	CCL7	ENST00000378569	166,502	3.09E+04
291	KLK5	ENST00000336334	166,451	1.60E+03
292	Q8WYN8	ENST00000344561	166,422	3.12E+04
293	SIRT2	ENST00000249396	166,393	3.04E+04
294	ISLR2	ENST00000360956	166,393	3.13E+04
295	HIST1H2BC	ENST00000314332	166,331	3.15E+04

296	TCEAL4	ENST00000372629	166,281	3.16E+04
297	CCDC114	ENST00000355682	166,266	1.51E+03
298	SLC25A15	ENST00000379523	166,256	3.17E+04
299	TESC	ENST00000335209	166,246	2.71E+03
300	C6orf65	ENST00000370748	166,197	3.19E+04
301	NP_694583.1	ENST00000295226	166,193	3.19E+04
302	CDK2AP1	ENST00000261692	166,112	2.77E+03
303	ELMOD1	ENST00000265840	166,102	2.71E+02
304	NEDD9	ENST00000379433	166,055	3.23E+04
305	DEGS1	ENST00000323699	165,979	6.17E+01
306	TRDC	ENST00000390477	165,925	3.28E+04
307	GLO1	ENST00000373365	165,899	6.21E+02
308	FLOT1	ENST00000376385	165,838	3.30E+04
309	Q6ZSQ7	ENST00000330476	165,791	3.95E+03
310	TTY11	ENST00000253470	165,791	3.32E+04
311	OR2T29	ENST00000328570	165,791	3.32E+04
312	HHLA2	ENST00000357759	165,791	3.32E+04
313	OR2T5	ENST00000366473	165,791	3.32E+04
314	PFKP	ENST00000381188	165,791	3.32E+04
315	HPS4	ENST00000312736	165,764	3.33E+04
316	HNRPR	ENST00000374612	165,754	4.32E+03
317	ADFP	ENST00000276914	165,751	5.35E+02
318	YIF1B	ENST00000339413	165,746	6.38E+01
319	GLI3	ENST00000265526	165,746	5.09E+02
320	COL5A1	ENST00000371815	165,746	7.06E+02
321	RASEF	ENST00000376447	165,746	7.06E+02
322	RNF6	ENST00000339626	165,746	8.85E+02
323	ALG5	ENST00000239891	165,746	5.08E+03
324	DGKA	ENST00000331886	165,746	1.05E+04
325	FBXW12	ENST00000296438	165,746	2.66E+04
326	MRPL36	ENST00000382647	165,746	2.97E+04
327	KYNU	ENST00000264170	165,746	3.33E+04
328	AZGP1	ENST00000292401	165,746	3.33E+04

329	NP_001017987.1	ENST00000292586	165,746	3.33E+04
330	MLLT10	ENST00000307729	165,746	3.33E+04
331	Q96FU4	ENST00000328202	165,746	3.33E+04
332	SNRPB	ENST00000336808	165,746	3.33E+04
333	RUFY2	ENST00000342616	165,746	3.33E+04
334	OMA1	ENST00000371226	165,746	3.33E+04
335	CTNNA3	ENST00000373735	165,746	3.33E+04
336	HES2	ENST00000377836	165,746	3.33E+04
337	A4D0Y5	ENST00000378768	165,746	3.33E+04
338	IL11RA	ENST00000378817	165,746	3.33E+04
339	SNRPB	ENST00000381342	165,746	3.33E+04
340	ARD1B	ENST00000286794	165,727	1.77E+04
341	HNRPR	ENST00000374614	165,701	4.36E+03
342	HNRPR	ENST00000374616	165,675	4.39E+03
343	VPS72	ENST00000368892	165,662	7.98E+03
344	CYP4Z1	ENST00000334194	165,645	3.37E+04
345	RAPGEF4	ENST00000264111	165,623	1.18E+03
346	C12orf52	ENST00000299731	165,623	3.38E+04
347	TM7SF4	ENST00000297581	165,604	5.29E+03
348	KIR2DL1	ENST00000339924	165,544	3.40E+04
349	PPP2R2A	ENST00000315985	165,505	3.42E+04
350	RPGR	ENST00000378505	165,501	1.17E+03
351	TMEM77	ENST00000369761	165,419	3.44E+04
352	RIC8A	ENST00000325207	165,416	3.45E+04
353	RXFP2	ENST00000298386	165,382	6.33E+01
354	CYP2C19	ENST00000371321	165,336	3.47E+04
355	C9orf86	ENST00000290079	165,319	3.48E+04
356	NCL	ENST00000356936	165,315	3.48E+04
357	NXT1	ENST00000254998	165,311	3.48E+04
358	CDC27	ENST00000066544	165,285	2.08E+01
359	RIC8A	ENST00000382752	165,259	3.50E+04
360	NCL	ENST00000322723	165,215	2.92E+03
361	ZSCAN2	ENST00000334141	165,188	3.52E+04

362	Q8N1W0	ENST00000325649	165,156	3.54E+04
363	TACR1	ENST00000305249	165,148	9.81E+01
364	TMEM77	ENST00000286692	165,146	3.54E+04
365	HIST1H2AL	ENST00000377352	165,088	3.56E+04
366	Q9H1T4	ENST00000379864	165,039	3.56E+04
367	CYP1A2	ENST00000343932	165,035	1.67E+03
368	HTR3A	ENST00000355556	165,017	3.58E+04
369	CCDC110	ENST00000307588	164,887	3.63E+04
370	IGFBP3	ENST00000381086	164,824	3.65E+04
371	Q6ZWH7	ENST00000374795	164,819	3.65E+04
372	GSN	ENST00000373807	164,808	3.66E+04
373	Q8N287	ENST00000355927	164,789	3.67E+04
374	GABARAPL3	ENST00000312365	164,786	8.48E+03
375	Q4G0P5	ENST00000321991	164,786	3.67E+04
376	FKHL18	ENST00000322577	164,786	3.67E+04
377	C21orf77	ENST00000334165	164,786	3.67E+04
378	Q5JXA8	ENST00000381628	164,786	3.67E+04
379	SH3BGR	ENST00000333634	164,769	4.64E+03
380	ZNF514	ENST00000295208	164,766	9.85E+03
381	P2RY4	ENST00000374519	164,757	1.74E+03
382	CKM	ENST00000221476	164,755	3.68E+04
383	PHACTR1	ENST00000379348	164,753	3.68E+04
384	C14orf103	ENST00000261834	164,655	1.19E+02
385	HSD17B4	ENST00000256216	164,645	1.45E+04
386	Q2TVT4	ENST00000377517	164,612	3.73E+04
387	Q2TVT4	ENST00000377549	164,612	3.73E+04
388	Q2TVT4	ENST00000377550	164,612	3.73E+04
389	SARS	ENST00000369923	164,603	3.68E+00
390	WDR16	ENST00000299764	164,603	4.79E+02
391	DCPS	ENST00000263579	164,603	4.93E+02
392	COL5A1	ENST00000371815	164,603	7.06E+02
393	IAG2	ENST00000373338	164,603	7.06E+02
394	RASEF	ENST00000376447	164,603	7.06E+02

395	ANXA4	ENST00000355054	164,603	1.49E+03
396	GABRR2	ENST00000229606	164,603	6.75E+03
397	RWDD2	ENST00000369724	164,603	2.91E+04
398	RWDD2	ENST00000014761	164,603	3.73E+04
399	MRPL32	ENST00000223324	164,603	3.73E+04
400	SARS	ENST00000234677	164,603	3.73E+04
401	CCDC74A	ENST00000295171	164,603	3.73E+04
402	CCDC74B	ENST00000310463	164,603	3.73E+04
403	ATP8A1	ENST00000340904	164,603	3.73E+04
404	APP	ENST00000355226	164,603	3.73E+04
405	DRD1IP	ENST00000368555	164,603	3.73E+04
406	SARS	ENST00000369920	164,603	3.73E+04
407	C9orf58	ENST00000372301	164,603	3.73E+04
408	TRAPPC3	ENST00000373159	164,603	3.73E+04
409	TKTL1	ENST00000369915	164,574	4.24E+03
410	FGF7P2	ENST00000332473	164,556	3.75E+04
411	ENTPD1	ENST00000371206	164,535	3.76E+04
412	RTN4	ENST00000317610	164,427	3.39E+03
413	SH3BGR	ENST00000380637	164,393	3.81E+04
414	ATG10	ENST00000282185	164,307	3.84E+04
415	LRFN2	ENST00000338305	164,301	1.71E+03
416	BTF3	ENST00000380591	164,297	3.85E+04
417	CDK5RAP2	ENST00000373942	164,248	3.82E+04
418	CTR9	ENST00000361944	164,243	6.81E+01
419	ZNF143	ENST00000299606	164,189	4.38E+03
420	CD244	ENST00000368032	164,073	3.93E+04
421	ASCL1	ENST00000266744	164,071	5.35E+02
422	GLDC	ENST00000321612	164,044	3.94E+04
423	SHANK1	ENST00000338916	163,951	3.98E+04
424	GPR31	ENST00000239583	163,947	3.98E+04
425	KCNK18	ENST00000334549	163,931	9.11E+03
426	CCDC96	ENST00000310085	163,925	2.37E+03
427	Q2TVT3	ENST00000377614	163,866	4.01E+04

428	UBE2E1	ENST00000306627	163,838	5.97E+03
429	GRIA1	ENST00000285900	163,805	4.04E+04
430	ASB4	ENST00000257621	163,797	4.04E+04
431	WBSCR17	ENST00000333538	163,782	3.89E+02
432	Q9P1L9	ENST00000315478	163,782	4.05E+04
433	ZNF441	ENST00000357901	163,782	4.05E+04
434	THEM5	ENST00000368817	163,782	4.05E+04
435	hCG_18385	ENST00000376856	163,782	4.05E+04
436	IGLV3-9	ENST00000390316	163,782	4.05E+04
437	MRPL52	ENST00000355151	163,764	1.31E+04
438	AMY2B	ENST00000330330	163,737	4.06E+04
439	TUSC3	ENST00000351598	163,737	4.06E+04
440	H2BFS	ENST00000380263	163,725	2.26E+03
441	CCR2	ENST00000292301	163,704	4.08E+04
442	TH1L	ENST00000344018	163,669	4.11E+03
443	NM_018652.4	ENST00000268079	163,666	4.09E+04
444	NFATC2	ENST00000371564	163,636	4.11E+04
445	SLC38A3	ENST00000341160	163,617	8.11E+03
446	Q9HAI8	ENST00000302857	163,614	4.11E+04
447	OAZ1	ENST00000322297	163,612	2.52E+04
448	RAD51L3	ENST00000345766	163,594	4.12E+04
449	SPDYC	ENST00000377185	163,585	4.13E+04
450	SCARA5	ENST00000301906	163,584	4.13E+04
451	SETDB2	ENST00000258672	163,573	4.13E+04
452	CSH2	ENST00000336844	163,572	4.13E+04
453	ZNF597	ENST00000301744	163,514	4.15E+04
454	MST1	ENST00000383728	163,504	4.16E+04
455	LAD1	ENST00000367314	163,456	4.18E+04
456	TMPRSS3	ENST00000380399	163,416	4.19E+04
457	SMARCA2	ENST00000382183	163,398	4.20E+04
458	MUSK	ENST00000374447	163,372	4.21E+04
459	Q2VIK4	ENST00000310962	163,356	4.22E+04
460	FPR1	ENST00000304748	163,349	3.40E+03

461	FHAD1	ENST00000314740	163,303	4.24E+04
462	EPC1	ENST00000375093	163,284	4.25E+04
463	EYA3	ENST00000373864	163,239	4.27E+04
464	C4orf21	ENST00000264370	163,226	4.27E+04
465	PXMP3	ENST00000357039	163,213	1.93E+04
466	Q6IEE8	ENST00000361112	163,175	4.30E+04
467	H2BFS	ENST00000361105	163,158	2.51E+03
468	PPA1	ENST00000373232	163,111	4.32E+04
469	ZDHHC11	ENST00000283441	163,091	4.33E+04
470	TNNI1	ENST00000367312	163,054	4.35E+04
471	CCL20	ENST00000358813	163,054	4.35E+04
472	TMEM69	ENST00000290802	163,037	1.81E+03
473	FER1L3	ENST00000359263	163,037	4.35E+04
474	C20orf4	ENST00000320849	163,025	4.36E+04
475	KRTAP9-3	ENST00000377719	163,017	2.15E+04
476	ARL6IP2	ENST00000332337	163,013	4.36E+04
477	SH2D3A	ENST00000245908	163,001	4.37E+04
478	C20orf4	ENST00000373932	162,997	4.37E+04
479	FAM25	ENST00000340853	162,996	4.37E+04
480	FAM25A	ENST00000357539	162,996	4.37E+04
481	DIAPH1	ENST00000389057	162,941	3.60E+02
482	C1orf162	ENST00000369718	162,929	4.40E+04
483	CD53	ENST00000271324	162,927	4.82E+01
484	DDX46	ENST00000389367	162,908	4.41E+04
485	MRPS2	ENST00000241600	162,907	4.41E+04
486	TMEM69	ENST00000372025	162,874	1.86E+03
487	GPR161	ENST00000271357	162,858	4.43E+04
488	MTHFSD	ENST00000322911	162,784	4.46E+04
489	ACTL7B	ENST00000277242	162,777	4.47E+04
490	NDUFA10	ENST00000307300	162,777	4.47E+04
491	ICA1	ENST00000317367	162,777	4.47E+04
492	RNF126	ENST00000340092	162,777	4.47E+04
493	GRM7	ENST00000357716	162,777	4.47E+04

494	Q6ZRI9	ENST00000361041	162,777	4.47E+04
495	OAT	ENST00000368845	162,769	4.47E+04
496	MRPL52	ENST00000311892	162,743	4.48E+04
497	PNPO	ENST00000225573	162,741	4.48E+04
498	C9orf53	ENST00000310702	162,731	4.49E+04
499	FABP3	ENST00000373713	162,685	4.36E+04
500	ENO1	ENST00000358874	162,669	4.51E+04
501	HCK	ENST00000262651	162,663	8.56E+03
502	CLEC4M	ENST00000327325	162,657	4.52E+04
503	GPR161	ENST00000367838	162,652	4.52E+04
504	NPL	ENST00000258317	162,648	4.52E+04
505	NM_203423	ENST00000318930	162,612	1.05E+03
506	ADHFE1	ENST00000276576	162,578	2.59E+00
507	RABGAP1L	ENST00000367690	162,558	4.56E+04
508	NM_203423	ENST00000382541	162,554	1.06E+03
509	ELK4	ENST00000289703	162,529	4.58E+04
510	AOC2	ENST00000253799	162,515	1.10E+04
511	ARHGAP21	ENST00000376408	162,493	4.59E+04
512	OPN4	ENST00000372071	162,478	7.57E+03
513	ENO1	ENST00000234590	162,464	4.60E+04
514	PGS1	ENST00000335081	162,464	4.60E+04
515	HCK	ENST00000375852	162,444	4.61E+04
516	KRT4	ENST00000293774	162,428	4.63E+03
517	DENND1A	ENST00000373620	162,417	4.63E+04
518	CTBP2	ENST00000334808	162,356	4.65E+04
519	ATXN2L	ENST00000359153	162,336	4.66E+04
520	SULT1A3	ENST00000338971	162,327	4.67E+04
521	CWF19L1	ENST00000370386	162,317	1.57E+01
522	SMPDL3A	ENST00000368440	162,317	6.68E+02
523	ZNF570	ENST00000330173	162,317	1.11E+03
524	CCL23	ENST00000293280	162,317	2.18E+03
525	SHANK1	ENST00000293441	162,317	2.18E+03
526	HTR2B	ENST00000258400	162,317	2.95E+03

527	LIG1	ENST00000263274	162,317	3.76E+03
528	CCT2	ENST00000299300	162,317	5.32E+03
529	CDK4	ENST00000257904	162,317	9.26E+03
530	LILRA2	ENST00000251376	162,317	4.67E+04
531	LILRA2	ENST00000251377	162,317	4.67E+04
532	F12	ENST00000253496	162,317	4.67E+04
533	KRT15	ENST00000254043	162,317	4.67E+04
534	ATP2C2	ENST00000262429	162,317	4.67E+04
535	DNAJC5B	ENST00000276570	162,317	4.67E+04
536	WDR90	ENST00000293880	162,317	4.67E+04
537	SERF2	ENST00000337861	162,317	4.67E+04
538	ASS1	ENST00000352480	162,317	4.67E+04
539	C10orf137	ENST00000356792	162,317	4.67E+04
540	SMARCD3	ENST00000356800	162,317	4.67E+04
541	NP_849155.2	ENST00000362026	162,317	4.67E+04
542	FCRL1	ENST00000368175	162,317	4.67E+04
543	NP_056263.1	ENST00000370147	162,317	4.67E+04
544	LOC647839	ENST00000370516	162,317	4.67E+04
545	ASS1	ENST00000372393	162,317	4.67E+04
546	MRRF	ENST00000373723	162,317	4.67E+04
547	PSMD5	ENST00000373920	162,317	4.67E+04
548	PKHD1L1	ENST00000378402	162,317	4.67E+04
549	PRPS2	ENST00000380663	162,317	4.67E+04
550	RAD17	ENST00000380777	162,317	4.67E+04
551	RNF170	ENST00000319073	162,287	4.68E+04
552	NIPSNAP1	ENST00000216121	162,272	1.33E+01
553	CHKA	ENST00000265689	162,272	1.46E+04
554	CYP2C9	ENST00000260682	162,262	4.70E+04
555	AGXT2L2	ENST00000323594	162,212	4.72E+04
556	GBGT1	ENST00000372040	162,138	4.75E+04
557	RNMT	ENST00000262173	162,133	4.76E+04
558	PRPF18	ENST00000378572	162,128	6.44E+03
559	TCF24	ENST00000340798	162,101	4.77E+04

560	C20orf57	ENST00000278979	162,095	4.77E+04
561	Q6ZS29	ENST00000376246	162,069	4.38E+04
562	IBRDC3	ENST00000373456	162,068	1.26E+03
563	CER1	ENST00000380911	162,063	1.10E+03
564	EFCAB5	ENST00000320856	162,063	4.79E+04
565	Q9Y2A2	ENST00000380161	162,051	4.79E+04
566	CCDC50	ENST00000282358	162,023	4.74E+02
567	DOCK2	ENST00000256935	162,005	1.04E+03
568	ZBTB38	ENST00000321464	162,002	4.82E+04
569	C9orf41	ENST00000376830	161,964	4.83E+04
570	CALML3	ENST00000315238	161,936	4.85E+04
571	Q2VIQ3	ENST00000330584	161,919	7.51E+03
572	CTNNA3	ENST00000373744	161,915	6.29E+02
573	ZBTB41	ENST00000329696	161,903	4.86E+04
574	IL3	ENST00000296870	161,857	4.88E+04
575	CPB2	ENST00000181383	161,852	2.77E+02
576	XR_017783.1	ENST00000377951	161,825	4.90E+04
577	IL3	ENST00000379197	161,805	7.38E+03
578	MLXIPL	ENST00000345114	161,805	4.91E+04
579	NP_775906.1	ENST00000316401	161,772	2.19E+04
580	NDEL1	ENST00000299734	161,772	2.67E+04
581	ATG3	ENST00000283290	161,772	4.93E+04
582	Q9H383	ENST00000287450	161,772	4.93E+04
583	RECQL5	ENST00000340830	161,772	4.93E+04
584	RABGAP1L	ENST00000367689	161,772	4.93E+04
585	OR10R2	ENST00000368152	161,772	4.93E+04
586	Q6ZS33	ENST00000370255	161,772	4.93E+04
587	NLRP6	ENST00000382637	161,772	4.93E+04
588	C3orf47	ENST00000383461	161,772	4.93E+04
589	TUBB4	ENST00000264071	161,767	3.88E+04
590	MRPS2	ENST00000371785	161,738	4.94E+04
591	LMAN1	ENST00000251047	161,716	4.95E+04
592	CD1C	ENST00000368169	161,704	4.96E+04

593	URM1	ENST00000372848	161,622	5.00E+04
594	CENPI	ENST00000372927	161,583	2.52E+03
595	DPY19L4	ENST00000342856	161,583	1.61E+04
596	PTPN4	ENST00000263708	161,547	4.86E+03
597	PCMT1	ENST00000367384	161,538	6.93E+03
598	IGLV5-52	ENST00000390289	161,463	3.37E+04
599	APLP1	ENST00000221891	161,445	4.15E+03
600	ME2	ENST00000321341	161,436	1.49E+03
601	TAC1	ENST00000319273	161,319	1.50E+02
602	ZMYND12	ENST00000372565	161,267	2.54E+02
603	DCTN3	ENST00000259632	161,174	1.69E+02
604	WDR25	ENST00000335290	161,174	7.50E+02
605	PLCG2	ENST00000359376	161,174	8.77E+02
606	INSL5	ENST00000304526	161,174	3.65E+03
607	Q6PJF6	ENST00000284676	161,174	3.90E+03
608	DHRS1	ENST00000288111	161,174	4.01E+03
609	SS18L1	ENST00000343379	161,174	4.02E+03
610	GARS	ENST00000265296	161,174	4.54E+03
611	DECR1	ENST00000220764	161,174	8.97E+03
612	SALL4	ENST00000217086	161,174	2.48E+04
613	SSBP1	ENST00000265304	161,174	2.57E+04
614	PRKD2	ENST00000291281	161,174	4.53E+04
615	GOT2	ENST00000245206	161,168	4.41E+03
616	WDR27	ENST00000333572	161,166	3.65E+03
617	CCNG2	ENST00000354403	161,127	5.35E+03
618	CRELD1	ENST00000295982	161,031	3.30E+02
619	HMGN3	ENST00000344726	161,025	1.04E+04
620	IFIT3	ENST00000371818	160,983	1.96E+04
621	C1orf162	ENST00000343534	160,966	1.17E+04
622	C10orf91	ENST00000321248	160,951	3.53E+04
623	OPN3	ENST00000366554	160,922	6.34E+03
624	EIF2AK3	ENST00000303236	160,881	2.14E+04
625	TMOD3	ENST00000308580	160,862	3.36E+02

626	MLLT10	ENST00000377091	160,784	3.44E+03
627	SMAD9	ENST00000379826	160,769	2.95E+03
628	Q71RC1	ENST00000382818	160,767	2.22E+03
629	TREH	ENST00000264029	160,767	3.13E+03
630	KLHL12	ENST00000367258	160,767	3.95E+03
631	Q9P1L8	ENST00000361252	160,767	2.98E+04
632	CCDC64	ENST00000257583	160,602	3.40E+03
633	TRBV18	ENST00000390392	160,602	2.43E+04
634	SLC12A6	ENST00000354181	160,462	4.02E+03
635	FAM21B	ENST00000374359	160,338	2.54E+03
636	DEC1	ENST00000374016	160,277	3.24E+03
637	Q8NFD4	ENST00000334373	160,235	3.44E+03
638	BICD1	ENST00000281474	160,228	9.88E+02
639	CLDN15	ENST00000308344	160,222	3.28E+03
640	PAN3	ENST00000380958	160,187	2.01E+02
641	TRNT1	ENST00000280591	160,121	1.93E+02
642	FADS6	ENST00000310226	160,104	3.35E+03
643	NSMAF	ENST00000038176	160,075	1.82E+04
644	PRDM7	ENST00000296682	160,055	3.74E+04
645	TMEM126B	ENST00000358867	160,031	3.45E+01
646	SVEP1	ENST00000374463	160,031	3.48E+01
647	FMO4	ENST00000367749	160,031	1.49E+02
648	ALLC	ENST00000252505	160,031	2.93E+02
649	CHEK2	ENST00000382580	160,031	8.36E+02
650	MLSTD1	ENST00000182377	160,031	3.10E+03
651	FAIM	ENST00000338446	160,031	3.40E+03
652	ARHGAP9	ENST00000340423	160,031	4.17E+03
653	DDX59	ENST00000331314	160,031	5.71E+03
654	ACTC1	ENST00000290378	160,031	1.48E+04
655	IFIT1	ENST00000371804	160,031	4.67E+04
656	C18orf34	ENST00000383095	160,031	4.68E+04
657	MBNL2	ENST00000376685	160,013	4.99E+02
658	SLITRK4	ENST00000356928	159,942	1.94E+03

659	TMEM45A	ENST00000323523	159,903	1.30E+03
660	TMEM69	ENST00000290802	159,819	1.81E+03
661	HOXA5	ENST00000222726	159,776	1.04E+03
662	IZUMO1	ENST00000332955	159,762	3.14E+02
663	SLC25A14	ENST00000339231	159,762	3.81E+03
664	TJP2	ENST00000377245	159,739	4.01E+04
665	TLR2	ENST00000260010	159,692	4.66E+03
666	SYN2	ENST00000341648	159,689	3.98E+03
667	TMEM69	ENST00000372025	159,659	1.86E+03
668	LRRN2	ENST00000367177	159,639	1.08E+04
669	GPD2	ENST00000310454	159,606	6.03E+01
670	PLAU	ENST00000372764	159,601	5.42E+03
671	SLFNL1	ENST00000359345	159,535	4.65E+03
672	GRPR	ENST00000380289	159,293	5.00E+04
673	NECAP2	ENST00000375568	159,253	9.32E+02
674	IGF2R	ENST00000356956	159,231	1.26E+04
675	FMO5	ENST00000254090	159,191	2.86E+03
676	CDH15	ENST00000289746	159,113	4.05E+03
677	IL12RB2	ENST00000262345	159,092	4.61E+03
678	NUAK2	ENST00000367157	158,976	2.99E+03
679	ASXL1	ENST00000375689	158,974	2.13E+03
680	FMO1	ENST00000367750	158,971	4.99E+03
681	SH2B1	ENST00000359285	158,928	4.79E+03
682	POLH	ENST00000372236	158,908	7.47E+03
683	GNG11	ENST00000248564	158,888	8.21E+02
684	FLJ16369	ENST00000250479	158,888	2.16E+03
685	GPR77	ENST00000257267	158,888	2.16E+03
686	ATP5S	ENST00000245448	158,888	3.36E+03
687	CPB1	ENST00000282957	158,888	4.21E+03
688	SERPINI2	ENST00000264677	158,888	4.23E+03
689	JAM2	ENST00000312957	158,888	4.23E+03
690	CASC1	ENST00000320267	158,888	4.23E+03
691	AASS	ENST00000358954	158,888	5.75E+03

692	PSTPIP1	ENST00000379595	158,888	8.61E+03
693	CDC2	ENST00000373811	158,843	3.95E+03
694	RASGEF1B	ENST00000264400	158,795	1.92E+04
695	SMNDC1	ENST00000369603	158,758	1.47E+00
696	Q71RC1	ENST00000382818	158,758	2.22E+03
697	MLNR	ENST00000218721	158,758	4.98E+03
698	ZP1	ENST00000278853	158,758	3.81E+04
699	Q9HAC4	ENST00000206466	158,758	4.21E+04
700	H2BFS	ENST00000380263	158,672	2.26E+03
701	ALS2	ENST00000264276	158,632	1.20E+04
702	USP44	ENST00000258499	158,517	3.39E+02
703	SLC35A4	ENST00000323146	158,503	1.13E+04
704	ZNF474	ENST00000296600	158,488	2.72E+04
705	TP53	ENST00000269305	158,446	3.09E+04
706	CCDC96	ENST00000310085	158,424	2.37E+03
707	RRH	ENST00000317735	158,423	4.62E+03
708	DUSP22	ENST00000344450	158,416	5.85E+03
709	NP_060358.2	ENST00000331849	158,386	1.73E+04
710	OPTC	ENST00000367222	158,361	4.66E+03
711	MBOAT5	ENST00000261407	158,335	3.67E+01
712	CSMD1	ENST00000335551	158,335	4.70E+03
713	CRNN	ENST00000271835	158,333	6.86E+03
714	SFRS11	ENST00000370950	158,189	1.17E+02
715	TBCK	ENST00000273980	158,132	1.53E+02
716	H2BFS	ENST00000361105	158,123	2.51E+03
717	C2orf17	ENST00000273048	158,121	1.49E+04
718	CACNA1A	ENST00000360228	158,103	6.72E+02
719	TTC19	ENST00000261647	158,101	6.44E+03
720	PENK	ENST00000314922	158,064	9.77E+02
721	FAM21B	ENST00000374359	158,064	2.54E+03
722	C15orf48	ENST00000344300	158,057	4.96E+03
723	ITM2B	ENST00000378565	158,009	1.70E+00
724	RAE1	ENST00000371242	157,893	1.41E+02

725	PCDH15	ENST00000373965	157,763	5.41E+03
726	Q6ZU72	ENST00000369791	157,753	4.21E+04
727	Q96IR2	ENST00000357015	157,753	4.69E+04
728	Q9UHZ6	ENST00000360740	157,753	4.70E+04
729	FTSJ2	ENST00000332565	157,749	2.44E+04
730	CSTF2	ENST00000372974	157,745	5.06E+02
731	PCM1	ENST00000325083	157,745	6.29E+02
732	CYP2F1	ENST00000331105	157,745	1.34E+03
733	CLCN5	ENST00000376088	157,745	3.36E+03
734	CLCN5	ENST00000376091	157,745	3.36E+03
735	TBCB	ENST00000221855	157,745	7.24E+03
736	PAOX	ENST00000278060	157,745	7.69E+03
737	COPB2	ENST00000333188	157,745	8.81E+03
738	SAKS1	ENST00000294119	157,745	1.38E+04
739	NP_689579.3	ENST00000368011	157,745	1.78E+04
740	TCF4	ENST00000354452	157,745	2.58E+04
741	HDAC2	ENST00000368632	157,745	3.36E+04
742	GKN2	ENST00000328895	157,745	3.67E+04
743	WDR64	ENST00000366552	157,699	1.26E+03
744	QDPR	ENST00000382282	157,649	1.06E+04
745	QPRT	ENST00000219771	157,524	6.52E+03
746	LHX4	ENST00000263726	157,511	9.80E+03
747	CA8	ENST00000317995	157,455	1.06E+04
748	GOLGA4	ENST00000361924	157,387	3.52E+03
749	SLC5A6	ENST00000380196	157,326	6.85E+03
750	FAT	ENST00000260147	157,317	1.18E+03
751	POLDIP3	ENST00000252115	157,306	8.31E+03
752	EXDL2	ENST00000193422	157,291	8.55E+03
753	CDH10	ENST00000382239	157,279	1.98E+03
754	OC90	ENST00000254627	157,262	2.02E+04
755	BEXL1	ENST00000372691	157,234	3.64E+03
756	ELAC2	ENST00000338034	157,201	7.75E+03
757	DHX37	ENST00000308736	157,148	6.66E+03

758	WRNIP1	ENST00000380773	157,123	4.03E+04
759	CCDC60	ENST00000327554	157,122	6.09E+03
760	UGP2	ENST00000337130	157,122	2.64E+04
761	Q8NH96	ENST00000268912	157,115	3.69E+03
762	COL25A1	ENST00000333642	157,035	4.10E+04
763	NFATC1	ENST00000329101	157,008	7.81E+03
764	C6orf162	ENST00000229570	156,968	1.17E+01
765	VAX1	ENST00000369206	156,906	1.51E+03
766	HGS	ENST00000329138	156,772	4.96E+02
767	ETV2	ENST00000379026	156,748	4.01E+03
768	S100A14	ENST00000368702	156,723	1.31E+04
769	KBTBD10	ENST00000284669	156,664	6.45E+03
770	GDF10	ENST00000224605	156,619	7.14E+02
771	C1orf49	ENST00000319416	156,602	3.45E+01
772	UNC50	ENST00000357765	156,602	6.27E+01
773	FGL1	ENST00000221204	156,602	3.47E+02
774	MND1	ENST00000240488	156,602	5.80E+02
775	HPS6	ENST00000370027	156,602	1.01E+03
776	RNF17	ENST00000381927	156,602	1.40E+03
777	RDBP	ENST00000375429	156,602	1.45E+03
778	ATP5S	ENST00000245448	156,602	3.36E+03
779	CLCN5	ENST00000376088	156,602	3.36E+03
780	CLCN5	ENST00000376091	156,602	3.36E+03
781	HPS6	ENST00000299238	156,602	4.17E+03
782	SQRDL	ENST00000260324	156,602	6.71E+03
783	TMEM108	ENST00000321871	156,602	1.97E+04
784	DOCK2	ENST00000256935	156,568	1.04E+03
785	RTN4	ENST00000317610	156,543	3.39E+03
786	CCDC64	ENST00000257583	156,536	3.40E+03
787	GIMAP4	ENST00000255945	156,536	9.48E+03
788	MLLT10	ENST00000377091	156,468	3.44E+03
789	ATP10A	ENST00000356865	156,384	4.13E+01
790	SERPINE1	ENST00000223095	156,365	2.65E+04

791	FLOT2	ENST00000335362	156,358	3.07E+04
792	GEMIN5	ENST00000285873	156,353	1.91E+04
793	GOLGA4	ENST00000361924	156,345	3.52E+03
794	VBP1	ENST00000286428	156,303	1.02E+04
795	C1orf74	ENST00000294811	156,301	1.00E+04
796	MCF2	ENST00000370578	156,298	1.93E+03
797	KIAA1012	ENST00000283351	156,252	8.98E+03
798	GBE1	ENST00000264326	156,183	8.44E+03
799	BEXL1	ENST00000372691	156,172	3.64E+03
800	CENTG1	ENST00000328568	156,167	7.08E+03
801	Q8NH96	ENST00000268912	156,108	3.69E+03
802	HS3ST2	ENST00000261374	156,056	1.38E+04
803	Q8NGG1	ENST00000313276	156,012	4.48E+04
804	AFG3L2	ENST00000269143	155,994	8.95E+03
805	WWC1	ENST00000265293	155,992	1.14E+04
806	PSMD7	ENST00000219313	155,985	4.62E+03
807	RARRES1	ENST00000237696	155,955	6.78E+01
808	NUMBL	ENST00000252891	155,921	2.77E+02
809	FAM83G	ENST00000388995	155,917	2.57E+03
810	LRRC30	ENST00000383467	155,901	9.60E+03
811	ACTN1	ENST00000193403	155,828	8.51E+02
812	NES	ENST00000368223	155,805	2.50E+03
813	SLC25A15	ENST00000379534	155,776	5.11E+00
814	AICDA	ENST00000229335	155,743	1.51E+03
815	Q6ZSQ7	ENST00000330476	155,743	3.95E+03
816	KLHL12	ENST00000367258	155,743	3.95E+03
817	NUDT8	ENST00000376693	155,743	3.16E+04
818	CAB39L	ENST00000355854	155,722	2.04E+02
819	TMEM16B	ENST00000327087	155,564	7.92E+03
820	HCCS	ENST00000321143	155,498	1.03E+04
821	MERTK	ENST00000295408	155,489	2.95E+04
822	SNX6	ENST00000362031	155,483	8.82E+03
823	CASP8	ENST00000343290	155,459	1.30E+02

824	C16orf61	ENST00000219400	155,459	7.27E+02
825	C16orf73	ENST00000293936	155,459	7.27E+02
826	HPS6	ENST00000370027	155,459	1.01E+03
827	ATP5B	ENST00000262030	155,459	1.47E+03
828	MAPKAPK5	ENST00000202788	155,459	1.54E+03
829	BPIL1	ENST00000170150	155,459	4.17E+03
830	HPS6	ENST00000299238	155,459	4.17E+03
831	SHC3	ENST00000375831	155,459	4.17E+03
832	NP_064597.1	ENST00000268130	155,459	8.08E+03
833	RAP1GDS1	ENST00000339360	155,459	8.08E+03
834	RSPO3	ENST00000368317	155,459	8.08E+03
835	NPPB	ENST00000376468	155,459	8.08E+03
836	DCP1A	ENST00000294241	155,459	9.25E+03
837	C12orf60	ENST00000330828	155,459	1.07E+04
838	TBRG1	ENST00000375005	155,459	1.27E+04
839	C20orf67	ENST00000372409	155,459	2.34E+04
840	FAM26D	ENST00000368596	155,452	4.17E+03
841	FAM26D	ENST00000368596	155,452	4.17E+03
842	TMEM106C	ENST00000256686	155,451	2.16E+04
843	TACR2	ENST00000373306	155,417	2.49E+04
844	TNFAIP8L3	ENST00000327536	155,399	1.52E+03
845	MCCC2	ENST00000340941	155,373	8.21E+03
846	C5AR1	ENST00000355085	155,316	9.86E+02
847	TMBIM4	ENST00000286424	155,311	8.31E+03
848	SELK	ENST00000231908	155,309	1.30E+04
849	KIAA1815	ENST00000339450	155,284	8.35E+03
850	HNRPR	ENST00000374612	155,263	4.32E+03
851	HNRPR	ENST00000374614	155,214	4.36E+03
852	NP_775813.1	ENST00000280800	155,206	9.21E+03
853	HNRPR	ENST00000374616	155,189	4.39E+03
854	CTSD	ENST00000382005	155,184	8.50E+03
855	C8B	ENST00000371237	155,166	1.13E+03
856	GOT2	ENST00000245206	155,162	4.41E+03

857	BLMH	ENST00000261714	155,124	1.76E+04
858	ZSCAN20	ENST00000326544	155,118	1.32E+04
859	BCCIP	ENST00000368759	155,052	8.71E+03
860	ZRANB2	ENST00000340681	155,046	8.94E+03
861	NP_116012.2	ENST00000260011	155,021	1.75E+03
862	EDG4	ENST00000358804	154,997	2.29E+02
863	DUXA	ENST00000376239	154,984	1.19E+04
864	GPR141	ENST00000334425	154,974	5.02E+02
865	PSMD7	ENST00000219313	154,917	4.62E+03
866	KRT4	ENST00000293774	154,898	4.63E+03
867	FAM117A	ENST00000240364	154,897	2.82E+03
868	TBCK	ENST00000273980	154,883	1.53E+02
869	TBCK	ENST00000273980	154,883	1.53E+02
870	SH3BGR	ENST00000333634	154,883	4.64E+03
871	BRD2	ENST00000374825	18,512	8.49E+02
872	MSI2	ENST00000284073	18,039	7.64E+03
873	KLC3	ENST00000337446	17,832	9.43E+03
874	MRPL41	ENST00000371443	17,832	9.43E+03
875	ADAMTS6	ENST00000381052	17,749	1.03E+04
876	SLC25A15	ENST00000338625	17,728	1.05E+04
877	PPP2CB	ENST00000221138	17,657	6.90E+03
878	ASPH	ENST00000389214	17,397	1.46E+04
879	DQX1	ENST00000272440	17,291	7.34E+02
880	ZUBR1	ENST00000375219	17,196	1.79E+04
881	TRIM67	ENST00000366653	17,182	2.93E+03
882	ZUBR1	ENST00000375225	17,122	1.93E+04
883	ZUBR1	ENST00000375267	17,098	1.98E+04
884	GOLGA6	ENST00000290438	16,981	2.22E+04
885	NP_061122.4	ENST00000300576	16,981	2.22E+04
886	CDRT1	ENST00000354433	16,962	2.27E+04
887	EPB41L5	ENST00000331393	16,833	2.58E+04
888	SH3YL1	ENST00000309148	16,775	8.01E+03
889	CNTNAP3	ENST00000323947	16,764	2.76E+04

890	CNTNAP3B	ENST00000377561	16,764	2.76E+04
891	PSG2	ENST00000244295	16,756	2.79E+04
892	RMND1	ENST00000367303	16,737	7.82E+03
893	ATP13A2	ENST00000341676	16,717	2.90E+04
894	CPNE9	ENST00000383832	16,705	2.93E+04
895	LRRC8D	ENST00000337338	16,657	2.35E+03
896	NP_001032302.1	ENST00000301896	16,657	3.07E+04
897	TBC1D20	ENST00000382121	16,605	3.24E+04
898	Q6ZNQ2	ENST00000381466	16,602	3.25E+04
899	ZNF545	ENST00000319849	16,587	1.68E+03
900	PGLYRP4	ENST00000368739	16,535	3.47E+04
901	NEIL2	ENST00000284503	16,522	5.33E+03
902	RDH10	ENST00000240285	16,519	9.28E+02
903	WDR69	ENST00000373666	16,505	6.46E+02
904	WDR69	ENST00000373666	16,505	6.46E+02
905	SOHLH2	ENST00000379881	16,494	7.51E+03
906	FLOT1	ENST00000376389	16,458	7.61E+03
907	IL18	ENST00000280357	16,411	4.26E+03
908	GLDC	ENST00000381336	16,388	4.01E+04
909	COL6A3	ENST00000295550	16,378	4.05E+04
910	PLEKHO1	ENST00000025469	16,346	7.29E+01
911	IGSF21	ENST00000251296	16,346	3.77E+02
912	MORN3	ENST00000355329	16,346	4.13E+02
913	RNF6	ENST00000339626	16,346	8.85E+02
914	Q6ZP14	ENST00000378347	16,346	8.85E+02
915	ANKRD37	ENST00000335174	16,346	8.99E+02
916	WDR63	ENST00000355948	16,346	1.16E+03
917	GPR77	ENST00000257267	16,346	2.16E+03
918	GPR177	ENST00000354777	16,346	2.50E+03
919	C3orf25	ENST00000326085	16,346	1.23E+04
920	MYOZ1	ENST00000359322	16,346	2.16E+04
921	AKAP4	ENST00000358526	16,346	3.58E+04
922	MYL2	ENST00000228841	16,346	4.02E+04

923	EBNA1BP2	ENST00000236051	16,346	4.18E+04
924	CCT7	ENST00000258091	16,346	4.18E+04
925	ATF2	ENST00000264110	16,346	4.18E+04
926	FCN3	ENST00000270879	16,346	4.18E+04
927	REG3G	ENST00000272324	16,346	4.18E+04
928	WDR63	ENST00000294664	16,346	4.18E+04
929	TGIF2LY	ENST00000321217	16,346	4.18E+04
930	RAD1	ENST00000325577	16,346	4.18E+04
931	FCN3	ENST00000354982	16,346	4.18E+04
932	Q6ZRL6	ENST00000356374	16,346	4.18E+04
933	PPIA	ENST00000358455	16,346	4.18E+04
934	ARV1	ENST00000366655	16,346	4.18E+04
935	C10orf90	ENST00000368674	16,346	4.18E+04
936	C10orf137	ENST00000368813	16,346	4.18E+04
937	PLEKHO1	ENST00000369126	16,346	4.18E+04
938	WDR63	ENST00000370596	16,346	4.18E+04
939	ZWINT	ENST00000373940	16,346	4.18E+04
940	AKAP4	ENST00000376064	16,346	4.18E+04
941	IFNA4	ENST00000380222	16,346	4.18E+04
942	TGIF2LY	ENST00000383049	16,346	4.18E+04
943	ZF36	ENST00000341191	16,338	4.21E+04
944	SETDB2	ENST00000317257	16,334	1.63E+03
945	LOC653698	ENST00000243148	16,328	4.25E+04
946	GIYD2	ENST00000344620	16,328	4.25E+04
947	IFNA17	ENST00000380214	16,297	4.38E+04
948	GMPR	ENST00000259727	16,285	4.43E+04
949	BIRC2	ENST00000227758	16,272	4.49E+04
950	TSGA13	ENST00000354969	16,203	4.80E+04
951	ARAF	ENST00000377039	16,181	4.91E+04
952	RSRC2	ENST00000331738	16,135	3.72E+01
953	RND2	ENST00000225973	16,054	8.60E+03
954	Q9UHT8	ENST00000355239	16,032	4.95E+04
955	SCIN	ENST00000297029	16,004	1.03E+04

956	SCGN	ENST00000377961	15,993	4.21E+03
957	FNDC1	ENST00000297267	15,819	4.47E+04
958	NP_775734.1	ENST00000389613	15,788	5.52E+03
959	BAG2	ENST00000370693	15,734	5.68E+03
960	DRG1	ENST00000331457	15,726	8.75E+01
961	APBB1IP	ENST00000376236	15,687	1.73E+04
962	SAMD1	ENST00000269724	15,629	8.18E+02
963	DKK1	ENST00000373970	15,579	1.86E+04
964	MYO1E	ENST00000288235	15,576	2.23E+04
965	DENND1B	ENST00000367396	15,573	8.23E+03
966	LOH12CR1	ENST00000314565	15,508	8.67E+03
967	SNX11	ENST00000359238	16.53	3.49E+04
968	TKTL1	ENST00000217905	16.46	3.73E+04
969	SPCS1	ENST00000233025	15.8	5.55E+03

Supplemental Table 6: putative target genes for miR-143 identified with TargetScan

#	Target gene	Representative transcript	Total context+ score	Aggregate P
1	DENND1B	NM_001195215	-0.67	0.46
2	VASH1	NM_014909	-0.65	0.61
3	SLC30A8	NM_001172811	-0.60	0.50
4	ABL2	NM_001136000	-0.59	0.57
5	TTPA	NM_000370	-0.59	0.67
6	SLC25A15	NM_014252	-0.52	0.79
7	AKAP6	NM_004274	-0.48	0.76
8	ATG2B	NM_018036	-0.47	0.27
9	DLG3	NM_001166278	-0.45	0.42
10	ZNF583	NM_001159860	-0.45	0.12
11	HK2	NM_000189	-0.43	0.39
12	FOSL2	NM_005253	-0.43	0.43
13	EARS2	NM_001083614	-0.42	0.17
14	GIGYF2	NM_001103146	-0.41	0.85
15	ARHGAP26	NM_001135608	-0.41	0.50
16	ATP11C	NM_001010986	-0.40	0.26
17	GOLM1	NM_016548	-0.40	< 0.1
18	FADS6	NM_178128	-0.39	< 0.1
19	PAN3	NM_175854	-0.37	0.15
20	DIP2B	NM_173602	-0.37	0.39
21	UBE2E3	NM_006357	-0.37	0.11
22	IGFBP5	NM_000599	-0.36	0.79
23	NFATC1	NM_006162	-0.36	0.36
24	C13orf33	NM_032849	-0.36	0.18
25	MYO3A	NM_017433	-0.35	< 0.1
26	SECISBP2L	NM_001193489	-0.35	0.36
27	NPR3	NM_000908	-0.33	0.79
28	LMO4	NM_006769	-0.33	0.71
29	CACNA1A	NM_000068	-0.33	0.36
30	NRSN1	NM_080723	-0.32	0.11

31	PSME4	NM_014614	-0.32	< 0.1
32	PCMT1	NM_005389	-0.32	0.11
33	SIX4	NM_017420	-0.31	0.39
34	CALN1	NM_001017440	-0.31	0.21
35	NECAP1	NM_015509	-0.30	0.54
36	CCDC58	NM_001017928	-0.30	0.12
37	SFMBT2	NM_001018039	-0.29	0.11
38	GDF10	NM_004962	-0.29	0.37
39	BRD2	NM_001113182	-0.29	0.28
40	PLEKHG2	NM_022835	-0.29	< 0.1
41	HIPK2	NM_001113239	-0.29	0.68
42	MCF2	NM_001099855	-0.29	0.20
43	WWC3	NM_015691	-0.28	0.36
44	SP7	NM_001173467	-0.28	0.22
45	CRELD1	NM_001031717	-0.28	0.78
46	MAPK7	NM_002749	-0.28	0.78
47	RSRC2	NM_023012	-0.28	0.11
48	UBE2E1	NM_001202476	-0.28	0.18
49	NCBP1	NM_002486	-0.27	< 0.1
50	HTR7	NM_000872	-0.27	< 0.1
51	TOR1AIP2	NM_022347	-0.27	0.61
52	XK	NM_021083	-0.27	0.11
53	TARDBP	NM_007375	-0.27	0.44
54	ABCC4	NM_005845	-0.27	0.11
55	ARID3B	NM_006465	-0.27	0.58
56	TUB	NM_003320	-0.26	0.35
57	ZBTB44	NM_014155	-0.26	0.79
58	CRLF3	NM_015986	-0.26	0.36
59	KLF5	NM_001730	-0.26	< 0.1
60	PTPN11	NM_002834	-0.25	0.43
61	SOBP	NM_018013	-0.25	0.24
62	RNF6	NM_005977	-0.24	0.11
63	STXBP1	NM_001032221	-0.24	0.11

64	ADD3	NM_001121	-0.24	0.11
65	SCAMP4	NM_079834	-0.24	0.11
66	OTUD4	NM_001102653	-0.24	0.54
67	CALM1	NM_006888	-0.24	0.11
68	AFF1	NM_001166693	-0.23	0.73
69	FAM71F1	NM_032599	-0.23	< 0.1
70	LARP4	NM_001170803	-0.23	0.36
71	KRAS	NM_004985	-0.23	0.82
72	HTR2C	NM_000868	-0.23	0.24
73	CHST10	NM_004854	-0.23	< 0.1
74	RGSL1	NM_001137669	-0.23	0.11
75	TAOK2	NM_004783	-0.22	0.12
76	GABARAPL1	NM_031412	-0.22	0.11
77	ATP10B	NM_025153	-0.22	< 0.1
78	MLLT3	NM_004529	-0.22	0.11
79	FMN1	NM_001103184	-0.22	0.30
80	DCAKD	NM_001128631	-0.22	0.11
81	USP45	NM_001080481	-0.22	0.76
82	FAM60A	NM_001135811	-0.22	0.36
83	BBS9	NM_001033604	-0.22	0.29
84	MIP	NM_012064	-0.21	0.11
85	ZNF236	NM_007345	-0.21	< 0.1
86	C20orf11	NM_017896	-0.21	0.49
87	GFPT2	NM_005110	-0.21	< 0.1
88	AP2B1	NM_001030006	-0.21	0.17
89	SLC35F1	NM_001029858	-0.20	0.21
90	PAPD5	NM_001040284	-0.20	0.40
91	PLEKHM3	NM_001080475	-0.20	0.32
92	FAM111A	NM_001142519	-0.20	< 0.1
93	TPM3	NM_001043351	-0.20	0.81
94	EPT1	NM_033505	-0.19	0.38
95	FXYD3	NM_001136007	-0.19	0.13
96	SERPINE1	NM_000602	-0.19	0.12

97	SCAF8	NM_014892	-0.19	0.11
98	ADCYAP1R1	NM_001118	-0.19	0.24
99	CREBL2	NM_001310	-0.19	0.11
100	EPG5	NM_020964	-0.19	< 0.1
101	LOXL4	NM_032211	-0.18	0.34
102	ARL15	NM_019087	-0.18	0.11
103	PC	NM_000920	-0.18	0.36
104	EGLN1	NM_022051	-0.18	0.11
105	HHLA2	NM_007072	-0.18	0.11
106	SSH2	NM_033389	-0.18	0.24
107	NUAK2	NM_030952	-0.17	0.78
108	ENSA	NM_207043	-0.17	0.11
109	TMOD2	NM_001142885	-0.17	0.42
110	CCDC149	NM_001130726	-0.17	0.11
111	TCF20	NM_005650	-0.17	0.11
112	TMEM167B	NM_020141	-0.17	0.11
113	MYBL2	NM_002466	-0.17	0.26
114	PLAU	NM_001145031	-0.17	0.11
115	SLC16A2	NM_006517	-0.17	0.73
116	DAPK1	NM_004938	-0.16	0.11
117	ZHX3	NM_015035	-0.16	0.21
118	SYT3	NM_001160328	-0.16	0.11
119	DDAH1	NM_001134445	-0.16	0.37
120	TPM4	NM_001145160	-0.16	0.29
121	MARCH3	NM_178450	-0.15	0.15
122	DCX	NM_000555	-0.15	0.16
123	TSPAN14	NM_001128309	-0.15	0.29
124	UPF1	NM_002911	-0.15	0.72
125	DNAJC3	NM_006260	-0.15	0.26
126	IGF2R	NM_000876	-0.15	0.19
127	ASH1L	NM_018489	-0.15	0.26
128	FAM108B1	NM_001025780	-0.15	0.26
129	PDIA6	NM_005742	-0.15	0.26

130	WNT5B	NM_030775	-0.15	0.21
131	VPS37A	NM_001145152	-0.14	0.33
132	SLC7A11	NM_014331	-0.14	0.13
133	CACNA1C	NM_000719	-0.14	0.46
134	CBX5	NM_001127321	-0.14	0.52
135	SVEP1	NM_153366	-0.14	0.36
136	RPS6KL1	NM_031464	-0.14	0.26
137	SAMD8	NM_001174156	-0.14	0.30
138	PRKCE	NM_005400	-0.14	0.79
139	COL5A3	NM_015719	-0.14	0.26
140	UNC5A	NM_133369	-0.14	0.30
141	FAM117A	NM_030802	-0.14	0.11
142	BCORL1	NM_021946	-0.14	0.11
143	TXLNB	NM_153235	-0.14	0.24
144	TMEM132B	NM_052907	-0.13	0.11
145	CREBZF	NM_001039618	-0.13	0.53
146	CTGF	NM_001901	-0.13	0.26
147	KRT80	NM_001081492	-0.13	0.17
148	SIDT1	NM_017699	-0.13	< 0.1
149	MAF	NM_001031804	-0.13	0.79
150	AMOTL1	NM_130847	-0.13	0.27
151	TNP1	NM_003284	-0.13	0.26
152	MKL2	NM_014048	-0.13	0.46
153	LIMK2	NM_005569	-0.13	0.11
154	COL5A1	NM_000093	-0.12	0.15
155	ZNF148	NM_021964	-0.12	0.51
156	LIFR	NM_001127671	-0.12	0.34
157	PUS10	NM_144709	-0.12	0.26
158	ERCC6	NM_000124	-0.12	0.11
159	KANK4	NM_181712	-0.12	0.11
160	ARPC4-TTLL3	NM_001198793	-0.12	0.11
161	ZNF618	NM_133374	-0.12	0.55

162	ZNF275	NM_001080485	-0.12	0.42
163	LBH	NM_030915	-0.12	0.36
164	TAPBP	NM_003190	-0.12	0.12
165	C20orf111	NM_016470	-0.12	0.26
166	MARCKS	NM_002356	-0.11	0.26
167	MEX3C	NM_016626	-0.11	0.11
168	QKI	NM_206854	-0.11	0.71
169	VAMP7	NM_001145149	-0.11	0.26
170	GSR	NM_000637	-0.11	0.26
171	KLLN	NM_001126049	-0.11	0.11
172	MAPK1	NM_002745	-0.11	0.37
173	ARHGEF40	NM_018071	-0.11	0.11
174	HOXA7	NM_006896	-0.11	0.11
175	SLCO2A1	NM_005630	-0.10	0.16
176	LIMK1	NM_001204426	-0.10	0.75
177	SLC39A10	NM_001127257	-0.10	0.16
178	NDFIP1	NM_030571	-0.10	0.26
179	FAM100B	NM_182565	-0.10	0.26
180	SCN2B	NM_004588	-0.10	0.26
181	TRPS1	NM_014112	-0.10	0.47
182	RMND5A	NM_022780	-0.10	0.11
183	FUT4	NM_002033	-0.09	0.11
184	SLU7	NM_006425	-0.09	0.26
185	MBOAT2	NM_138799	-0.09	0.26
186	PGK1	NM_000291	-0.09	0.26
187	PDGFRA	NM_006206	-0.09	0.26
188	ARHGEF1	NM_004706	-0.09	0.33
189	CPEB2	NM_001177381	-0.09	0.26
190	GRHL2	NM_024915	-0.09	0.56
191	SLC25A25	NM_001006641	-0.09	0.26
192	RGAG1	NM_020769	-0.08	0.26
193	INO80D	NM_017759	-0.08	0.20
194	AP3M1	NM_012095	-0.08	0.26

195	DTNB	NM_021907	-0.08	0.11
196	SLC5A12	NM_178498	-0.08	0.26
197	CPLX2	NM_001008220	-0.08	0.13
198	RNF165	NM_152470	-0.08	0.53
199	TOB2	NM_016272	-0.07	0.11
200	TTYH3	NM_025250	-0.07	0.42
201	CPD	NM_001199775	-0.07	0.72
202	RICTOR	NM_152756	-0.07	0.15
203	PPP2R3A	NM_001190447	-0.07	0.26
204	C12orf68	NM_001013635	-0.07	0.11
205	FNDC3B	NM_001135095	-0.07	0.26
206	CBL	NM_005188	-0.06	< 0.1
207	SH3PXD2A	NM_014631	-0.06	0.84
208	RASAL2	NM_004841	-0.06	0.36
209	DIXDC1	NM_001037954	-0.06	0.15
210	FGF1	NM_000800	-0.06	0.39
211	FAM83F	NM_138435	-0.06	0.26
212	POU2F1	NM_001198783	-0.06	< 0.1
213	SPRY3	NM_005840	-0.05	0.18
214	CREB5	NM_001011666	-0.05	0.32
215	ATOH8	NM_032827	-0.05	0.34
216	HSPB7	NM_014424	-0.05	0.26
217	HDAC7	NM_001098416	-0.05	0.11
218	BCL2	NM_000633	-0.05	0.29
219	TSKU	NM_015516	-0.05	0.26
220	TRPC5	NM_012471	-0.05	0.26
221	SLC28A3	NM_001199633	-0.05	0.26
222	KPNA1	NM_002264	-0.05	0.15
223	ST8SIA4	NM_005668	-0.04	0.79
224	LARP1	NM_015315	-0.04	0.29
225	KCMF1	NM_020122	-0.04	0.27
226	SMAD3	NM_001145102	-0.04	0.13
227	PLXNA4	NM_020911	-0.04	0.37

228	CTC1	NM_025099	-0.04	0.11
229	TANC2	NM_025185	-0.04	0.35
230	ABHD2	NM_007011	-0.04	0.21
231	ENPP1	NM_006208	-0.04	0.26
232	SEZ6L	NM_001184773	-0.04	0.26
233	MLL2	NM_003482	-0.04	0.26
234	CACNA1E	NM_000721	-0.04	0.26
235	BBC3	NM_001127240	-0.04	0.26
236	MDGA1	NM_153487	-0.04	0.26
237	PTGS2	NM_000963	-0.04	0.26
238	ADD2	NM_001185054	-0.04	0.26
239	NRSN2	NM_024958	-0.04	0.11
240	SLC38A2	NM_018976	-0.03	0.42
241	USP46	NM_001134223	-0.03	0.36
242	LRRC8B	NM_001134476	-0.03	0.26
243	PLAGL2	NM_002657	-0.03	0.15
244	RALGPS1	NM_014636	-0.03	0.11
245	AMMECR1	NM_001025580	-0.03	0.11
246	ROD1	NM_001163788	-0.03	0.11
247	TET2	NM_001127208	-0.03	< 0.1
248	ANKRD33B	NM_001164440	-0.03	< 0.1
249	SLC22A3	NM_021977	-0.03	< 0.1
250	EEF2K	NM_013302	-0.03	0.34
251	SEC14L5	NM_014692	-0.03	0.34
252	SV2B	NM_001167580	-0.03	0.24
253	BSN	NM_003458	-0.03	0.16
254	RLIM	NM_016120	-0.03	0.25
255	RASSF6	NM_177532	-0.03	< 0.1
256	IQSEC1	NM_001134382	-0.03	0.11
257	FNDC5	NM_001171940	-0.03	0.26
258	LASP1	NM_006148	-0.02	0.36
259	MARK2	NM_001039469	-0.02	0.20
260	KLF13	NM_015995	-0.02	0.11

261	UCK2	NM_012474	-0.02	0.11
262	ESRRG	NM_001134285	-0.02	0.11
263	KCNJ10	NM_002241	-0.02	0.11
264	TMEM127	NM_001193304	-0.02	0.11
265	FAM123B	NM_152424	-0.02	0.35
266	KCNK10	NM_021161	-0.02	0.29
267	LSAMP	NM_002338	-0.02	0.29
268	FGD6	NM_018351	-0.02	0.15
269	IGF1R	NM_000875	-0.01	0.26
270	TNRC6C	NM_001142640	-0.01	0.26
271	DGKB	NM_004080	-0.01	0.26
272	EPB41	NM_001166005	-0.01	0.26
273	MPPED1	NM_001044370	-0.01	0.26
274	PTPN14	NM_005401	-0.01	0.26
275	SOX6	NM_001145811	-0.01	0.26
276	SESTD1	NM_178123	-0.01	0.26
277	CASK	NM_001126054	-0.01	0.26
278	COX18	NM_173827	-0.01	0.26
279	MFSD6	NM_017694	N/A	< 0.1

Supplemental Table 7: putative target genes for miR-143 based on TCGA data

#	Entrez Gene ID	HGNC ID	rho	p.val
1	GALM	130589	-0.318	1.80E-05
2	GEMIN8P4	492303	-0.280	1.66E-04
3	SNHG1	23642	-0.280	1.72E-04
4	SNHG3	8420	-0.275	2.23E-04
5	C6orf125	84300	-0.269	3.04E-04
6	FLJ39653	202020	-0.266	3.67E-04
7	ZNF587	84914	-0.263	4.31E-04
8	BCL2L14	79370	-0.263	4.33E-04
9	LOC150197	150197	-0.258	5.15E-04
10	MYL5	4636	-0.258	5.49E-04
11	ZNF679	168417	-0.254	6.59E-04
12	BRDT	676	-0.253	6.89E-04
13	C1orf111	284680	-0.252	7.02E-04
14	UNC13B	10497	-0.252	7.50E-04
15	LOC100131726	100131726	-0.251	7.41E-04
16	LRRC16A	55604	-0.250	8.38E-04
17	MCM9	254394	-	8.68E-

			0.249	04
18	NCRNA00107	283981	-<br>0.248	9.07E-<br>04
19	CCHCR1	54535	-<br>0.247	9.30E-<br>04
20	WDR52	55779	-<br>0.247	9.63E-<br>04
21	GLB1L3	112937	-<br>0.247	9.34E-<br>04
22	SLC36A2	153201	-<br>0.246	9.80E-<br>04
23	LRRC66	339977	-<br>0.245	1.03E-<br>03
24	CORO2A	7464	-<br>0.243	1.13E-<br>03
25	RPS24	6229	-<br>0.242	1.23E-<br>03
26	RSPH1	89765	-<br>0.241	1.24E-<br>03
27	SNHG8	100093630	-<br>0.240	1.36E-<br>03
28	MAP3K13	9175	-<br>0.239	1.38E-<br>03
29	IQCC	55721	-<br>0.237	1.51E-<br>03
30	KIAA0319	9856	-<br>0.237	1.48E-<br>03
31	?	100133144	-<br>0.237	1.53E-<br>03
32	PLCH1	23007	-<br>0.237	1.54E-<br>03
33	GOLGB1	2804	-<br>0.235	1.72E-<br>03
34	C8orf39	55472	-<br>0.234	1.76E-<br>03

35	CXCL17	284340	- 0.233	1.77E-03
36	RPL11	6135	- 0.232	1.96E-03
37	LOC143666	143666	- 0.231	1.99E-03
38	C1orf21	81563	- 0.231	2.05E-03
39	MDN1	23195	- 0.230	2.12E-03
40	DCDC2B	149069	- 0.226	2.53E-03
41	ZBTB3	79842	- 0.225	2.61E-03
42	HOXB6	3216	- 0.224	2.75E-03
43	LOC442454	442454	- 0.224	2.80E-03
44	ARHGAP32	9743	- 0.223	2.97E-03
45	LOC401052	401052	- 0.222	3.02E-03
46	ACPP	55	- 0.222	3.04E-03
47	CHD7	55636	- 0.221	3.20E-03
48	TPR	7175	- 0.221	3.22E-03
49	SNORD90	692206	- 0.221	3.18E-03
50	CTRC	11330	- 0.220	3.23E-03
51	CSMD3	114788	- 0.219	3.45E-03
52	GAS5	60674	-	3.57E-

			0.218	03
53	C8orf44	56260	-0.218	3.68E-03
54	DYDC1	143241	-0.217	3.73E-03
55	CUZD1	50624	-0.217	3.78E-03
56	LOC146880	146880	-0.216	3.91E-03
57	C2CD3	26005	-0.216	3.97E-03
58	TSC1	7248	-0.216	3.99E-03
59	HMBS	3145	-0.216	4.02E-03
60	PLEKHH1	57475	-0.215	4.10E-03
61	RPL34	6164	-0.215	4.13E-03
62	C11orf48	79081	-0.215	4.13E-03
63	FXYD3	5349	-0.215	4.16E-03
64	ASMT	438	-0.215	4.09E-03
65	GCNT7	140687	-0.214	4.24E-03
66	COX6C	1345	-0.213	4.43E-03
67	ARHGEF38	54848	-0.213	4.47E-03
68	NBPF7	343505	-0.212	4.56E-03
69	IVNS1ABP	10625	-0.212	4.76E-03

70	ACP6	51205	-<br>0.212	4.76E-<br>03
71	MORN1	79906	-<br>0.211	4.82E-<br>03
72	CASP5	838	-<br>0.211	4.82E-<br>03
73	DKFZP686I15217	401232	-<br>0.211	4.88E-<br>03
74	NCRNA00167	440072	-<br>0.210	4.94E-<br>03
75	RPL37A	6168	-<br>0.210	5.04E-<br>03
76	CYP4Z1	199974	-<br>0.210	5.03E-<br>03
77	CTTN	2017	-<br>0.210	5.16E-<br>03
78	PPARA	5465	-<br>0.210	5.17E-<br>03
79	ZNF692	55657	-<br>0.210	5.18E-<br>03
80	C12orf27	283460	-<br>0.209	5.22E-<br>03
81	C11orf49	79096	-<br>0.209	5.41E-<br>03
82	SNHG5	387066	-<br>0.208	5.56E-<br>03
83	RBM6	10180	-<br>0.207	5.72E-<br>03
84	C1orf126	200197	-<br>0.207	5.81E-<br>03
85	ARL16	339231	-<br>0.207	5.81E-<br>03
86	GUSBL1	387036	-<br>0.207	5.86E-<br>03
87	C2orf72	257407	-	5.93E-

			0.206	03
88	RPS10P7	376693	-<br>0.206	5.96E-<br>03
89	RPS17	6218	-<br>0.206	6.11E-<br>03
90	BARX2	8538	-<br>0.205	6.19E-<br>03
91	UQCRH	7388	-<br>0.205	6.20E-<br>03
92	BMS1	9790	-<br>0.205	6.36E-<br>03
93	PPEF2	5470	-<br>0.205	6.30E-<br>03
94	NANOS1	340719	-<br>0.204	6.41E-<br>03
95	CYP4Z2P	163720	-<br>0.204	6.39E-<br>03
96	LOC100132832	100132832	-<br>0.204	6.53E-<br>03
97	DNAJC5G	285126	-<br>0.204	6.47E-<br>03
98	SNTN	132203	-<br>0.204	6.52E-<br>03
99	TAS2R4	50832	-<br>0.203	6.61E-<br>03
100	RPL7	6129	-<br>0.203	6.74E-<br>03
101	HOXB5	3215	-<br>0.203	6.90E-<br>03
102	SNORA66	26782	-<br>0.202	6.88E-<br>03
103	INADL	10207	-<br>0.201	7.25E-<br>03
104	C2CD4B	388125	-<br>0.201	7.27E-<br>03

105	KCNK6	9424	-<br>0.201	7.27E-<br>03
106	KCNQ1OT1	10984	-<br>0.201	7.29E-<br>03
107	RAVER2	55225	-<br>0.201	7.31E-<br>03
108	SARS2	54938	-<br>0.201	7.37E-<br>03
109	RASSF7	8045	-<br>0.201	7.46E-<br>03
110	KIAA1530	57654	-<br>0.201	7.48E-<br>03
111	SPDYE7P	441251	-<br>0.200	7.54E-<br>03
112	FMO6P	388714	-<br>0.200	7.52E-<br>03
113	LOC645676	645676	-<br>0.200	7.63E-<br>03
114	CDK5RAP2	55755	-<br>0.200	7.71E-<br>03
115	SLC26A6	65010	-<br>0.200	7.78E-<br>03
116	RG9MTD3	158234	-<br>0.199	8.04E-<br>03
117	TBX3	6926	-<br>0.199	8.12E-<br>03
118	METTL3	56339	-<br>0.199	8.12E-<br>03
119	ZNF169	169841	-<br>0.199	8.14E-<br>03
120	FAM24B	196792	-<br>0.198	8.34E-<br>03
121	FAM186A	121006	-<br>0.197	8.46E-<br>03
122	SNORA27	619499	-	8.52E-

			0.197	03
123	MS4A10	341116	-<br>0.197	8.65E-<br>03
124	KIFC2	90990	-<br>0.197	8.67E-<br>03
125	SIM2	6493	-<br>0.197	8.72E-<br>03
126	LOC100131193	100131193	-<br>0.196	8.97E-<br>03
127	SNHG12	85028	-<br>0.196	9.04E-<br>03
128	LOC100129534	100129534	-<br>0.195	9.13E-<br>03
129	RPL23P8	222901	-<br>0.195	9.27E-<br>03
130	MYO18A	399687	-<br>0.195	9.39E-<br>03
131	PIGZ	80235	-<br>0.195	9.42E-<br>03
132	SMPD2	6610	-<br>0.195	9.48E-<br>03
133	TIGD1	200765	-<br>0.195	9.53E-<br>03
134	GEMIN7	79760	-<br>0.194	9.64E-<br>03
135	ANKZF1	55139	-<br>0.194	9.64E-<br>03
136	PRICKLE4	29964	-<br>0.194	9.64E-<br>03
137	LOC115110	115110	-<br>0.194	9.68E-<br>03
138	MGC16384	114130	-<br>0.194	9.61E-<br>03
139	SPAG5	10615	-<br>0.194	9.79E-<br>03

140	TTC39A	22996	-<br>0.194	9.85E-<br>03
141	C20orf106	200232	-<br>0.194	9.78E-<br>03
142	KIF9	64147	-<br>0.194	9.89E-<br>03
143	SNORA61	677838	-<br>0.194	9.82E-<br>03
144	SOD1	6647	-<br>0.193	1.01E-<br>02
145	NBR2	10230	-<br>0.193	1.02E-<br>02
146	MAP3K9	4293	-<br>0.193	1.02E-<br>02
147	NSUN7	79730	-<br>0.193	1.03E-<br>02
148	LOC100131434	100131434	-<br>0.192	1.04E-<br>02
149	NANOG	79923	-<br>0.192	1.04E-<br>02
150	TBC1D3	729873	-<br>0.192	1.05E-<br>02
151	LOC145837	145837	-<br>0.192	1.05E-<br>02
152	FAM106A	80039	-<br>0.192	1.05E-<br>02
153	CEACAM20	125931	-<br>0.192	1.05E-<br>02
154	CELSR3	1951	-<br>0.191	1.08E-<br>02
155	CCNO	10309	-<br>0.191	1.08E-<br>02
156	NHSL1	57224	-<br>0.191	1.11E-<br>02
157	GPRC5D	55507	-	1.10E-

			0.191	02
158	PLEKHA6	22874	-<br>0.190	1.15E-<br>02
159	NSUN5P2	260294	-<br>0.190	1.15E-<br>02
160	LOC157381	157381	-<br>0.190	1.15E-<br>02
161	MAPK15	225689	-<br>0.189	1.17E-<br>02
162	LOC284232	284232	-<br>0.189	1.18E-<br>02
163	SLC44A3	126969	-<br>0.189	1.18E-<br>02
164	RABGGTB	5876	-<br>0.189	1.18E-<br>02
165	C11orf90	387804	-<br>0.189	1.19E-<br>02
166	ACOT13	55856	-<br>0.189	1.21E-<br>02
167	IQCH	64799	-<br>0.188	1.22E-<br>02
168	LOC147727	147727	-<br>0.188	1.23E-<br>02
169	QPCTL	54814	-<br>0.187	1.27E-<br>02
170	BMS1P5	399761	-<br>0.187	1.27E-<br>02
171	NBAS	51594	-<br>0.187	1.27E-<br>02
172	STYK1	55359	-<br>0.187	1.27E-<br>02
173	ZYG11A	440590	-<br>0.187	1.28E-<br>02
174	C4orf19	55286	-<br>0.187	1.28E-<br>02

175	KIAA0368	23392	-<br>0.187	1.28E-<br>02
176	SLC10A5	347051	-<br>0.187	1.28E-<br>02
177	EPS8L3	79574	-<br>0.187	1.29E-<br>02
178	GSDMB	55876	-<br>0.187	1.30E-<br>02
179	SIK2	23235	-<br>0.187	1.30E-<br>02
180	CNPY1	285888	-<br>0.186	1.29E-<br>02
181	C17orf86	654434	-<br>0.186	1.31E-<br>02
182	SPDYE5	442590	-<br>0.186	1.33E-<br>02
183	POU2F1	5451	-<br>0.186	1.33E-<br>02
184	MSX2P1	55545	-<br>0.186	1.34E-<br>02
185	HSPC072	29075	-<br>0.186	1.34E-<br>02
186	C11orf65	160140	-<br>0.186	1.34E-<br>02
187	NAA40	79829	-<br>0.185	1.36E-<br>02
188	UBQLN4	56893	-<br>0.185	1.37E-<br>02
189	SNORA24	677809	-<br>0.185	1.36E-<br>02
190	ZNF432	9668	-<br>0.185	1.37E-<br>02
191	AFM	173	-<br>0.185	1.37E-<br>02
192	CARD14	79092	-	1.39E-

			0.185	02
193	TNIK	23043	-0.184	1.41E-02
194	AOX2P	344454	-0.184	1.43E-02
195	ATP5G1	516	-0.184	1.45E-02
196	SLC29A2	3177	-0.184	1.45E-02
197	DPPA2	151871	-0.183	1.45E-02
198	EPN3	55040	-0.183	1.46E-02
199	C22orf30	253143	-0.183	1.48E-02
200	OR1L8	138881	-0.183	1.50E-02
201	TADA2A	6871	-0.183	1.51E-02
202	EFCAB4A	283229	-0.183	1.51E-02
203	C16orf3	750	-0.182	1.51E-02
204	SURF6	6838	-0.182	1.52E-02
205	RNF183	138065	-0.182	1.52E-02
206	GAS2L3	283431	-0.182	1.53E-02
207	DDX31	64794	-0.182	1.53E-02
208	MCTP2	55784	-0.182	1.53E-02
209	PSPC1	55269	-0.182	1.55E-02

210	GLB1L2	89944	-0.182	1.55E-02
211	DKFZp686O24166	374383	-0.182	1.55E-02
212	LRRIQ4	344657	-0.182	1.54E-02
213	ADAM3A	1587	-0.182	1.57E-02
214	USH1C	10083	-0.181	1.58E-02
215	LOC100272217	100272217	-0.181	1.58E-02
216	GPRIN3	285513	-0.181	1.60E-02
217	FHIT	2272	-0.181	1.61E-02
218	LCOR	84458	-0.181	1.62E-02
219	ZFYVE27	118813	-0.181	1.62E-02
220	PPP1R3E	90673	-0.180	1.64E-02
221	FCAMR	83953	-0.180	1.63E-02
222	HOXB8	3218	-0.180	1.65E-02
223	COX7C	1350	-0.180	1.66E-02
224	UQCRB	7381	-0.180	1.66E-02
225	TSEN34	79042	-0.180	1.66E-02
226	SULT1C2	6819	-0.180	1.67E-02
227	ITPR3	3710	-	1.67E-

			0.180	02
228	CDC42BPG	55561	-<br>0.180	1.67E-<br>02
229	RPS23	6228	-<br>0.180	1.69E-<br>02
230	SERGEF	26297	-<br>0.180	1.69E-<br>02
231	RPL31	6160	-<br>0.180	1.69E-<br>02
232	MSGN1	343930	-<br>0.179	1.70E-<br>02
233	FGF8	2253	-<br>0.179	1.70E-<br>02
234	DET1	55070	-<br>0.179	1.71E-<br>02
235	C22orf27	150291	-<br>0.179	1.72E-<br>02
236	LOC338651	338651	-<br>0.179	1.72E-<br>02
237	TACC2	10579	-<br>0.179	1.73E-<br>02
238	AFMID	125061	-<br>0.179	1.74E-<br>02
239	DPP4	1803	-<br>0.179	1.75E-<br>02
240	TRIM10	10107	-<br>0.179	1.75E-<br>02
241	ZFP62	643836	-<br>0.179	1.76E-<br>02
242	APOBEC1	339	-<br>0.178	1.76E-<br>02
243	SNORD53	26796	-<br>0.178	1.75E-<br>02
244	LOC90246	90246	-<br>0.178	1.76E-<br>02

245	METTL12	751071	-<br>0.178	1.77E-<br>02
246	NPNT	255743	-<br>0.178	1.77E-<br>02
247	N4BP2	55728	-<br>0.178	1.78E-<br>02
248	CENPF	1063	-<br>0.178	1.78E-<br>02
249	S100A7	6278	-<br>0.178	1.78E-<br>02
250	LOC126536	126536	-<br>0.178	1.78E-<br>02
251	C9orf68	55064	-<br>0.178	1.80E-<br>02
252	SETDB1	9869	-<br>0.178	1.80E-<br>02
253	PLCB3	5331	-<br>0.178	1.80E-<br>02
254	ACYP1	97	-<br>0.178	1.80E-<br>02
255	TMPRSS11BNL	401136	-<br>0.178	1.80E-<br>02
256	BTBD8	284697	-<br>0.177	1.81E-<br>02
257	C2orf83	56918	-<br>0.177	1.82E-<br>02
258	TEP1	7011	-<br>0.177	1.84E-<br>02
259	RPL32P3	132241	-<br>0.177	1.85E-<br>02
260	TMCO6	55374	-<br>0.177	1.85E-<br>02
261	UQCRHL	440567	-<br>0.177	1.86E-<br>02
262	HIATL2	84278	-	1.88E-

			0.177	02
263	LHX4	89884	- 0.177	1.87E- 02
264	C1orf91	56063	- 0.176	1.90E- 02
265	GMDS	2762	- 0.176	1.91E- 02
266	SFRS15	57466	- 0.176	1.92E- 02
267	KIAA1984	84960	- 0.176	1.93E- 02
268	HRNR	388697	- 0.176	1.92E- 02
269	ZNF784	147808	- 0.175	1.96E- 02
270	LOC400752	400752	- 0.175	1.96E- 02
271	ANKHD1- EIF4EBP3	404734	- 0.175	1.98E- 02
272	ACACA	31	- 0.175	1.99E- 02
273	LOC440944	440944	- 0.175	1.99E- 02
274	CATSPER2	117155	- 0.175	1.99E- 02
275	ZBTB8B	728116	- 0.175	1.99E- 02
276	ZNF488	118738	- 0.175	1.99E- 02
277	CALML4	91860	- 0.175	2.00E- 02
278	ZNF789	285989	- 0.175	2.02E- 02
279	MAGI3	260425	- 0.174	2.03E- 02

280	PTPRH	5794	-<br>0.174	2.04E-<br>02
281	PAN2	9924	-<br>0.174	2.04E-<br>02
282	C14orf156	81892	-<br>0.174	2.04E-<br>02
283	VPS52	6293	-<br>0.174	2.05E-<br>02
284	PABPC1L2A	340529	-<br>0.174	2.06E-<br>02
285	OXER1	165140	-<br>0.174	2.07E-<br>02
286	TOMM20L	387990	-<br>0.174	2.07E-<br>02
287	FAM136B	387071	-<br>0.174	2.07E-<br>02
288	C4orf10	317648	-<br>0.174	2.08E-<br>02
289	CCL15	6359	-<br>0.174	2.08E-<br>02
290	FDPSL2A	619190	-<br>0.174	2.09E-<br>02
291	GCC2	9648	-<br>0.173	2.10E-<br>02
292	HEMK1	51409	-<br>0.173	2.13E-<br>02
293	LOC80054	80054	-<br>0.173	2.13E-<br>02
294	ZNF417	147687	-<br>0.173	2.15E-<br>02
295	MLL	4297	-<br>0.173	2.15E-<br>02
296	USP24	23358	-<br>0.173	2.17E-<br>02
297	C1orf159	54991	-	2.19E-

			0.172	02
298	LOC729609	729609	-<br>0.172	2.18E-<br>02
299	FKBPL	63943	-<br>0.172	2.19E-<br>02
300	TMEM143	55260	-<br>0.172	2.19E-<br>02
301	PTH	5741	-<br>0.172	2.19E-<br>02
302	LOC648691	648691	-<br>0.172	2.20E-<br>02
303	DUXA	503835	-<br>0.172	2.21E-<br>02
304	?	645851	-<br>0.172	2.21E-<br>02
305	C3orf27	23434	-<br>0.172	2.22E-<br>02
306	ALPK1	80216	-<br>0.172	2.24E-<br>02
307	MKI67	4288	-<br>0.172	2.24E-<br>02
308	BRS3	680	-<br>0.172	2.23E-<br>02
309	CKAP5	9793	-<br>0.171	2.26E-<br>02
310	ZXDC	79364	-<br>0.171	2.26E-<br>02
311	SNHG10	283596	-<br>0.171	2.26E-<br>02
312	HDHD3	81932	-<br>0.171	2.27E-<br>02
313	RBM5	10181	-<br>0.171	2.27E-<br>02
314	RGPD1	400966	-<br>0.171	2.28E-<br>02

315	UBXN7	26043	-0.171	2.28E-02
316	BMS1P4	729096	-0.171	2.29E-02
317	ZNF311	282890	-0.171	2.29E-02
318	ACRV1	56	-0.171	2.30E-02
319	LIMCH1	22998	-0.171	2.32E-02
320	CDK5RAP3	80279	-0.170	2.34E-02
321	LOC642852	642852	-0.170	2.36E-02
322	CDK11A	728642	-0.170	2.36E-02
323	AGBL4	84871	-0.170	2.36E-02
324	ZNF552	79818	-0.170	2.37E-02
325	DDX11L2	84771	-0.170	2.36E-02
326	S100P	6286	-0.170	2.38E-02
327	SF3B1	23451	-0.170	2.38E-02
328	DSC1	1823	-0.170	2.38E-02
329	GTF3C6	112495	-0.170	2.40E-02
330	LOC100129716	100129716	-0.170	2.40E-02
331	SPTAN1	6709	-0.170	2.40E-02
332	ZNF749	388567	-	2.42E-

			0.170	02
333	FRK	2444	-<br>0.169	2.43E-<br>02
334	USP54	159195	-<br>0.169	2.45E-<br>02
335	PDDC1	347862	-<br>0.169	2.46E-<br>02
336	RSPH6A	81492	-<br>0.169	2.45E-<br>02
337	NUP98	4928	-<br>0.169	2.47E-<br>02
338	PLCE1	51196	-<br>0.169	2.47E-<br>02
339	DNAH7	56171	-<br>0.169	2.48E-<br>02
340	TLN2	83660	-<br>0.169	2.49E-<br>02
341	SFI1	9814	-<br>0.169	2.50E-<br>02
342	LOC285359	285359	-<br>0.168	2.53E-<br>02
343	LOC285419	285419	-<br>0.168	2.53E-<br>02
344	DYX1C1	161582	-<br>0.168	2.55E-<br>02
345	RPS12	6206	-<br>0.168	2.55E-<br>02
346	TIAF1	9220	-<br>0.168	2.55E-<br>02
347	TMC1	117531	-<br>0.168	2.55E-<br>02
348	PDXDC2	283970	-<br>0.168	2.57E-<br>02
349	SLC35E2	728661	-<br>0.168	2.57E-<br>02

350	HNRNPA3P1	10151	-0.168	2.58E-02
351	C14orf79	122616	-0.168	2.59E-02
352	LOC643955	643955	-0.168	2.58E-02
353	LOC642826	642826	-0.167	2.61E-02
354	KIAA1244	57221	-0.167	2.61E-02
355	LOC729603	729603	-0.167	2.61E-02
356	OTUD3	23252	-0.167	2.61E-02
357	LENG9	94059	-0.167	2.62E-02
358	GALE	2582	-0.167	2.62E-02
359	VRK3	51231	-0.167	2.63E-02
360	LRRC37A4	55073	-0.167	2.63E-02
361	?	652919	-0.167	2.63E-02
362	CELF1	10658	-0.167	2.64E-02
363	CLDND2	125875	-0.167	2.64E-02
364	COX7B2	170712	-0.167	2.64E-02
365	ZNF479	90827	-0.167	2.64E-02
366	NOS2	4843	-0.167	2.66E-02
367	RBMY1B	378948	-	2.65E-

			0.167	02
368	UBR2	23304	-0.167	2.68E-02
369	LOC100133331	100133331	-0.166	2.69E-02
370	RNF168	165918	-0.166	2.71E-02
371	NASP	4678	-0.166	2.71E-02
372	C3orf62	375341	-0.166	2.72E-02
373	LOC648740	648740	-0.166	2.73E-02
374	OR7E156P	283491	-0.166	2.74E-02
375	ACCS	84680	-0.166	2.74E-02
376	C6orf124	653483	-0.166	2.74E-02
377	SMCR5	140771	-0.166	2.74E-02
378	RNF214	257160	-0.166	2.77E-02
379	NOP14	8602	-0.166	2.77E-02
380	TEC	7006	-0.166	2.77E-02
381	SPTLC2	9517	-0.166	2.78E-02
382	HMGN3	9324	-0.165	2.79E-02
383	WARS2	10352	-0.165	2.79E-02
384	ADM2	79924	-0.165	2.79E-02

385	FLJ42627	645644	-<br>0.165	2.79E-<br>02
386	OR52R1	119695	-<br>0.165	2.78E-<br>02
387	XPO5	57510	-<br>0.165	2.80E-<br>02
388	SPATA24	202051	-<br>0.165	2.81E-<br>02
389	NDUFA8	4702	-<br>0.165	2.83E-<br>02
390	HTR1D	3352	-<br>0.165	2.84E-<br>02
391	PKDREJ	10343	-<br>0.165	2.83E-<br>02
392	C6orf134	79969	-<br>0.165	2.85E-<br>02
393	RFX4	5992	-<br>0.165	2.86E-<br>02
394	C15orf17	57184	-<br>0.164	2.88E-<br>02
395	CCDC30	728621	-<br>0.164	2.89E-<br>02
396	TACC3	10460	-<br>0.164	2.91E-<br>02
397	SLC14A2	8170	-<br>0.164	2.94E-<br>02
398	ZNF226	7769	-<br>0.164	2.96E-<br>02
399	IQCB1	9657	-<br>0.163	2.98E-<br>02
400	TROAP	10024	-<br>0.163	2.99E-<br>02
401	PCNT	5116	-<br>0.163	2.99E-<br>02
402	DPAGT1	1798	-	3.00E-

			0.163	02
403	NSD1	64324	-<br>0.163	3.01E-<br>02
404	MSH5	4439	-<br>0.163	3.01E-<br>02
405	CCDC56	28958	-<br>0.163	3.02E-<br>02
406	SPTBN1	6711	-<br>0.163	3.03E-<br>02
407	C1orf104	284618	-<br>0.163	3.04E-<br>02
408	C6orf132	647024	-<br>0.163	3.04E-<br>02
409	AGAP5	729092	-<br>0.163	3.04E-<br>02
410	PDXDC1	23042	-<br>0.163	3.04E-<br>02
411	LOC100133612	100133612	-<br>0.163	3.05E-<br>02
412	C2orf68	388969	-<br>0.163	3.06E-<br>02
413	PEX7	5191	-<br>0.163	3.06E-<br>02
414	KIF13A	63971	-<br>0.163	3.07E-<br>02
415	C6orf208	80069	-<br>0.163	3.06E-<br>02
416	RPS27A	6233	-<br>0.162	3.08E-<br>02
417	EIF4A2	1974	-<br>0.162	3.09E-<br>02
418	OSCP1	127700	-<br>0.162	3.09E-<br>02
419	SNORD12B	100113393	-<br>0.162	3.08E-<br>02

420	KIAA0114	57291	-<br>0.162	3.10E-<br>02
421	TRIM66	9866	-<br>0.162	3.10E-<br>02
422	ACIN1	22985	-<br>0.162	3.10E-<br>02
423	NCRNA00095	283932	-<br>0.162	3.11E-<br>02
424	KRTAP3-3	85293	-<br>0.162	3.13E-<br>02
425	ZNF620	253639	-<br>0.162	3.14E-<br>02
426	TAS2R41	259287	-<br>0.162	3.14E-<br>02
427	LETMD1	25875	-<br>0.162	3.16E-<br>02
428	CBX8	57332	-<br>0.162	3.17E-<br>02
429	SETD4	54093	-<br>0.162	3.17E-<br>02
430	PPIP5K1	9677	-<br>0.162	3.18E-<br>02
431	CYP2R1	120227	-<br>0.161	3.19E-<br>02
432	F11R	50848	-<br>0.161	3.20E-<br>02
433	INSR	3643	-<br>0.161	3.20E-<br>02
434	C14orf128	84837	-<br>0.161	3.21E-<br>02
435	C14orf165	414767	-<br>0.161	3.22E-<br>02
436	SSU72	29101	-<br>0.161	3.23E-<br>02
437	CASP10	843	-	3.25E-

			0.161	02
438	DGAT1	8694	-<br>0.161	3.27E-<br>02
439	FAM138D	677784	-<br>0.160	3.29E-<br>02
440	LOC646851	646851	-<br>0.160	3.31E-<br>02
441	NIPAL1	152519	-<br>0.160	3.31E-<br>02
442	C9orf173	441476	-<br>0.160	3.31E-<br>02
443	DEPDC5	9681	-<br>0.160	3.33E-<br>02
444	AFG3L2	10939	-<br>0.160	3.33E-<br>02
445	NFRKB	4798	-<br>0.160	3.34E-<br>02
446	MYO7B	4648	-<br>0.160	3.35E-<br>02
447	C10orf110	55853	-<br>0.160	3.35E-<br>02
448	PABPN1	8106	-<br>0.160	3.35E-<br>02
449	ERCC6	2074	-<br>0.160	3.36E-<br>02
450	C8orf30A	51236	-<br>0.160	3.36E-<br>02
451	DSG4	147409	-<br>0.160	3.35E-<br>02
452	CMTM4	146223	-<br>0.160	3.36E-<br>02
453	ZNF833	401898	-<br>0.160	3.37E-<br>02
454	SLC9A4	389015	-<br>0.160	3.37E-<br>02

455	IAPP	3375	-<br>0.160	3.38E-<br>02
456	C17orf93	360205	-<br>0.160	3.38E-<br>02
457	TBC1D3H	729877	-<br>0.160	3.40E-<br>02
458	KCNJ3	3760	-<br>0.159	3.40E-<br>02
459	C8orf75	619351	-<br>0.159	3.40E-<br>02
460	C18orf16	147429	-<br>0.159	3.41E-<br>02
461	PPP1R1B	84152	-<br>0.159	3.41E-<br>02
462	MAS1	4142	-<br>0.159	3.40E-<br>02
463	RWDD2A	112611	-<br>0.159	3.41E-<br>02
464	LOC100190938	100190938	-<br>0.159	3.41E-<br>02
465	CHCHD8	51287	-<br>0.159	3.42E-<br>02
466	TM4SF20	79853	-<br>0.159	3.42E-<br>02
467	EXOSC2	23404	-<br>0.159	3.42E-<br>02
468	LIAS	11019	-<br>0.159	3.43E-<br>02
469	MYSM1	114803	-<br>0.159	3.45E-<br>02
470	GPR125	166647	-<br>0.159	3.45E-<br>02
471	SAMD12	401474	-<br>0.159	3.45E-<br>02
472	LGR4	55366	-	3.46E-

			0.159	02
473	FOXO3B	2310	-0.159	3.47E-02
474	GPR110	266977	-0.159	3.46E-02
475	TBC1D3C	414060	-0.159	3.48E-02
476	PFDN6	10471	-0.159	3.48E-02
477	BTBD18	643376	-0.159	3.47E-02
478	C1orf74	148304	-0.159	3.48E-02
479	KIAA1109	84162	-0.159	3.48E-02
480	SCAND2	54581	-0.159	3.49E-02
481	ZKSCAN3	80317	-0.159	3.50E-02
482	CDH3	1001	-0.158	3.52E-02
483	LOC93622	93622	-0.158	3.53E-02
484	LOC730101	730101	-0.158	3.53E-02
485	SSRP1	6749	-0.158	3.53E-02
486	SRRM2	23524	-0.158	3.54E-02
487	ADAT2	134637	-0.158	3.55E-02
488	LOC400891	400891	-0.158	3.55E-02
489	SERPINB6	5269	-0.158	3.55E-02

490	ATP5O	539	-0.158	3.56E-02
491	SAMD7	344658	-0.158	3.56E-02
492	AMY2B	280	-0.158	3.58E-02
493	RAG1AP1	55974	-0.158	3.58E-02
494	XKR9	389668	-0.158	3.59E-02
495	NMNAT1	64802	-0.158	3.60E-02
496	AIM1	202	-0.158	3.61E-02
497	KIAA0319L	79932	-0.158	3.62E-02
498	LRIG2	9860	-0.158	3.62E-02
499	GPR128	84873	-0.158	3.64E-02
500	RALGPS1	9649	-0.157	3.64E-02
501	YEATS2	55689	-0.157	3.65E-02
502	TEDDM1	127670	-0.157	3.65E-02
503	INTS3	65123	-0.157	3.66E-02
504	GUCA1B	2979	-0.157	3.67E-02
505	SNAR-I	100170222	-0.157	3.66E-02
506	ZNF829	374899	-0.157	3.67E-02
507	CUTA	51596	-	3.67E-

			0.157	02
508	LOC388152	388152	-<br>0.157	3.68E-<br>02
509	HOXB13	10481	-<br>0.157	3.68E-<br>02
510	XDH	7498	-<br>0.157	3.71E-<br>02
511	NSUN5P1	155400	-<br>0.157	3.72E-<br>02
512	ZNF841	284371	-<br>0.157	3.75E-<br>02
513	NANS	54187	-<br>0.156	3.77E-<br>02
514	PNKD	25953	-<br>0.156	3.77E-<br>02
515	ZSCAN16	80345	-<br>0.156	3.78E-<br>02
516	AGR2	10551	-<br>0.156	3.80E-<br>02
517	CEP192	55125	-<br>0.156	3.82E-<br>02
518	MED19	219541	-<br>0.156	3.82E-<br>02
519	SNORD4A	26773	-<br>0.156	3.81E-<br>02
520	C3orf35	339883	-<br>0.156	3.81E-<br>02
521	C4orf12	404201	-<br>0.156	3.82E-<br>02
522	DTX4	23220	-<br>0.156	3.86E-<br>02
523	HMGN2	3151	-<br>0.156	3.86E-<br>02
524	NKX6-2	84504	-<br>0.155	3.88E-<br>02

525	FOXO3	2309	-0.155	3.90E-02
526	ENOSF1	55556	-0.155	3.90E-02
527	TFRC	7037	-0.155	3.91E-02
528	C6orf26	401251	-0.155	3.91E-02
529	NPHS1	4868	-0.155	3.91E-02
530	RGPD3	653489	-0.155	3.92E-02
531	ZNF16	7564	-0.155	3.93E-02
532	KIF24	347240	-0.155	3.96E-02
533	SECISBP2	79048	-0.155	3.97E-02
534	MYL10	93408	-0.155	3.96E-02
535	TCEB3B	51224	-0.155	3.97E-02
536	LOC148413	148413	-0.155	3.98E-02
537	ODF2	4957	-0.155	3.99E-02
538	CDRT4	284040	-0.155	3.99E-02
539	ZBTB5	9925	-0.155	3.99E-02
540	MFSD3	113655	-0.155	4.01E-02
541	PXMP2	5827	-0.154	4.01E-02
542	SERHL	94009	-	4.02E-

			0.154	02
543	OSR2	116039	-<br>0.154	4.03E-<br>02
544	C4orf26	152816	-<br>0.154	4.02E-<br>02
545	DDB1	1642	-<br>0.154	4.05E-<br>02
546	CLPS	1208	-<br>0.154	4.04E-<br>02
547	WNK4	65266	-<br>0.154	4.06E-<br>02
548	KAZALD1	81621	-<br>0.154	4.07E-<br>02
549	HOOK2	29911	-<br>0.154	4.07E-<br>02
550	ANKRD36B	57730	-<br>0.154	4.09E-<br>02
551	RGPD8	727851	-<br>0.154	4.11E-<br>02
552	SNORD74	619498	-<br>0.154	4.10E-<br>02
553	ECHDC2	55268	-<br>0.154	4.11E-<br>02
554	TSTD1	100131187	-<br>0.154	4.11E-<br>02
555	NAPSA	9476	-<br>0.154	4.11E-<br>02
556	NCRNA00093	100188954	-<br>0.154	4.12E-<br>02
557	SNORD88A	692202	-<br>0.154	4.12E-<br>02
558	KDM1A	23028	-<br>0.154	4.14E-<br>02
559	FEZF1	389549	-<br>0.153	4.14E-<br>02

560	C21orf62	56245	-0.153	4.16E-02
561	ATP5L	10632	-0.153	4.17E-02
562	CARD16	114769	-0.153	4.17E-02
563	SNORA55	677834	-0.153	4.16E-02
564	RGPD4	285190	-0.153	4.17E-02
565	SIDT1	54847	-0.153	4.18E-02
566	TMEM125	128218	-0.153	4.19E-02
567	C17orf77	146723	-0.153	4.19E-02
568	HRSP12	10247	-0.153	4.20E-02
569	ABCA13	154664	-0.153	4.21E-02
570	SNORD18A	595098	-0.153	4.21E-02
571	TTC17	55761	-0.153	4.22E-02
572	TP53AIP1	63970	-0.153	4.21E-02
573	STX3	6809	-0.153	4.23E-02
574	SLC25A27	9481	-0.153	4.23E-02
575	IL22	50616	-0.153	4.22E-02
576	C21orf58	54058	-0.152	4.30E-02
577	FAM157A	728262	-	4.30E-

			0.152	02
578	NME6	10201	-0.152	4.30E-02
579	DMBT1	1755	-0.152	4.30E-02
580	POLQ	10721	-0.152	4.32E-02
581	TBR1	10716	-0.152	4.35E-02
582	PNN	5411	-0.152	4.36E-02
583	LOC127841	127841	-0.152	4.37E-02
584	C1orf226	400793	-0.152	4.38E-02
585	AGBL3	340351	-0.152	4.37E-02
586	TIGD2	166815	-0.152	4.39E-02
587	CTH	1491	-0.152	4.39E-02
588	RP1-177G6.2	286411	-0.152	4.40E-02
589	STAG3L3	442578	-0.152	4.42E-02
590	AGBL2	79841	-0.152	4.41E-02
591	PGM3	5238	-0.151	4.43E-02
592	PGAP1	80055	-0.151	4.43E-02
593	ZDHHC8P1	150244	-0.151	4.44E-02
594	OR1M1	125963	-0.151	4.43E-02

595	SLC30A9	10463	-<br>0.151	4.45E-<br>02
596	GPR126	57211	-<br>0.151	4.46E-<br>02
597	SDR9C7	121214	-<br>0.151	4.46E-<br>02
598	CCDC36	339834	-<br>0.151	4.49E-<br>02
599	KIAA1875	340390	-<br>0.151	4.49E-<br>02
600	TTC29	83894	-<br>0.151	4.48E-<br>02
601	SNHG4	724102	-<br>0.151	4.50E-<br>02
602	GAK	2580	-<br>0.151	4.51E-<br>02
603	RGPD6	729540	-<br>0.151	4.51E-<br>02
604	KRTAP5-1	387264	-<br>0.151	4.51E-<br>02
605	LNX1	84708	-<br>0.151	4.52E-<br>02
606	ZNF224	7767	-<br>0.151	4.53E-<br>02
607	LOC285501	285501	-<br>0.151	4.53E-<br>02
608	IDH1	3417	-<br>0.151	4.55E-<br>02
609	GNG13	51764	-<br>0.150	4.56E-<br>02
610	TTC31	64427	-<br>0.150	4.58E-<br>02
611	ZNF565	147929	-<br>0.150	4.58E-<br>02
612	NBPF4	148545	-	4.58E-

			0.150	02
613	FAM74A4	401508	-<br>0.150	4.58E-<br>02
614	MLH3	27030	-<br>0.150	4.59E-<br>02
615	CNFN	84518	-<br>0.150	4.60E-<br>02
616	FABP5	2171	-<br>0.150	4.62E-<br>02
617	PRR15L	79170	-<br>0.150	4.62E-<br>02
618	FUT4	2526	-<br>0.150	4.62E-<br>02
619	GLI4	2738	-<br>0.150	4.62E-<br>02
620	ASCC3	10973	-<br>0.150	4.62E-<br>02
621	LRTOMT	220074	-<br>0.150	4.62E-<br>02
622	CDSN	1041	-<br>0.150	4.64E-<br>02
623	UAP1	6675	-<br>0.150	4.64E-<br>02
624	LOC642846	642846	-<br>0.150	4.64E-<br>02
625	SLC35A3	23443	-<br>0.150	4.65E-<br>02
626	ZDHHC5	25921	-<br>0.150	4.66E-<br>02
627	IARS	3376	-<br>0.150	4.66E-<br>02
628	TBC1D3G	654341	-<br>0.150	4.66E-<br>02
629	BOD1L	259282	-<br>0.150	4.67E-<br>02

630	CCDC64B	146439	-0.150	4.67E-02
631	GSX1	219409	-0.150	4.66E-02
632	CA13	377677	-0.150	4.67E-02
633	LYZL4	131375	-0.150	4.66E-02
634	KIAA0100	9703	-0.150	4.68E-02
635	UBR4	23352	-0.150	4.69E-02
636	CNKSR1	10256	-0.150	4.69E-02
637	LOC100128288	100128288	-0.150	4.70E-02
638	KIAA2018	205717	-0.150	4.70E-02
639	MCM3APAS	114044	-0.150	4.71E-02
640	C1orf64	149563	-0.149	4.71E-02
641	DOPEY2	9980	-0.149	4.72E-02
642	CCNI2	645121	-0.149	4.73E-02
643	CDH7	1005	-0.149	4.72E-02
644	ACOX3	8310	-0.149	4.73E-02
645	CDRT15	146822	-0.149	4.74E-02
646	COPG2	26958	-0.149	4.75E-02
647	PIK3C2B	5287	-	4.76E-

			0.149	02
648	LOC100132287	100132287	-0.149	4.78E-02
649	KIAA1328	57536	-0.149	4.80E-02
650	LRRC56	115399	-0.149	4.81E-02
651	LOC100133161	100133161	-0.149	4.82E-02
652	KIAA0753	9851	-0.149	4.82E-02
653	EEF1G	1937	-0.149	4.83E-02
654	RASEF	158158	-0.149	4.83E-02
655	LQK1	642946	-0.149	4.86E-02
656	DHTKD1	55526	-0.148	4.88E-02
657	C1orf182	128229	-0.148	4.88E-02
658	LOC153684	153684	-0.148	4.92E-02
659	C15orf28	80035	-0.148	4.95E-02
660	RPS20	6224	-0.148	4.96E-02
661	TMPRSS7	344805	-0.148	4.95E-02
662	SLC26A8	116369	-0.148	4.96E-02
663	TATDN1	83940	-0.148	4.97E-02
664	C20orf70	140683	-0.148	4.97E-02

665	SF3B2	10992	-0.148	4.99E-02
666	ZNF700	90592	-0.148	4.99E-02
667	NBPF1	55672	-0.148	4.99E-02
668	IFT140	9742	-0.148	5.00E-02
669	RGPD5	84220	-0.148	5.00E-02
